# Cardiac-Specific Over-Expression of Epidermal Growth Factor Receptor 2 (ErbB2) Induces Pro-Survival Pathways and Hypertrophic Cardiomyopathy in Mice

**DOI:** 10.1371/journal.pone.0042805

**Published:** 2012-08-09

**Authors:** Polina Sysa-Shah, Yi Xu, Xin Guo, Frances Belmonte, Byunghak Kang, Djahida Bedja, Scott Pin, Noriko Tsuchiya, Kathleen Gabrielson

**Affiliations:** 1 Johns Hopkins University, School of Medicine, Department of Molecular and Comparative Pathobiology, Baltimore, Maryland, United States of America; 2 Drug Safety Evaluation, Drug Developmental Research Laboratories, Shionogi & Co., Ltd., Osaka, Japan; University of Pecs Medical School, Hungary

## Abstract

**Background:**

Emerging evidence shows that ErbB2 signaling has a critical role in cardiomyocyte physiology, based mainly on findings that blocking ErbB2 for cancer therapy is toxic to cardiac cells. However, consequences of high levels of ErbB2 activity in the heart have not been previously explored.

**Methodology/Principal Findings:**

We investigated consequences of cardiac-restricted over-expression of ErbB2 in two novel lines of transgenic mice. Both lines develop striking concentric cardiac hypertrophy, without heart failure or decreased life span. ErbB2 transgenic mice display electrocardiographic characteristics similar to those found in patients with Hypertrophic Cardiomyopathy, with susceptibility to adrenergic-induced arrhythmias. The hypertrophic hearts, which are 2–3 times larger than those of control littermates, express increased atrial natriuretic peptide and β-myosin heavy chain mRNA, consistent with a hypertrophic phenotype. Cardiomyocytes in these hearts are significantly larger than wild type cardiomyocytes, with enlarged nuclei and distinctive myocardial disarray. Interestingly, the over-expression of ErbB2 induces a concurrent up-regulation of multiple proteins associated with this signaling pathway, including EGFR, ErbB3, ErbB4, PI3K subunits p110 and p85, bcl-2 and multiple protective heat shock proteins. Additionally, ErbB2 up-regulation leads to an anti-apoptotic shift in the ratio of bcl-xS/xL in the heart. Finally, ErbB2 over-expression results in increased activation of the translation machinery involving S6, 4E-BP1 and eIF4E. The dependence of this hypertrophic phenotype on ErbB family signaling is confirmed by reduction in heart mass and cardiomyocyte size, and inactivation of pro-hypertrophic signaling in transgenic animals treated with the ErbB1/2 inhibitor, lapatinib.

**Conclusions/Significance:**

These studies are the first to demonstrate that increased ErbB2 over-expression in the heart can activate protective signaling pathways and induce a phenotype consistent with Hypertrophic Cardiomyopathy. Furthermore, our work suggests that in the situation where ErbB2 signaling contributes to cardiac hypertrophy, inhibition of this pathway may reverse this process.

## Introduction

ErbB2 (HER2/neu), a member of the EGFR family of receptor tyrosine kinases, first attracted attention after the discovery that this gene is amplified and over-expressed in a high percentage of breast cancers. The importance of ErbB2 signaling in cardiac physiology soon became evident by a discovery that some breast cancer patients treated with Trastuzumab (Herceptin, anti-ErbB2), an inhibitor of HER2 signaling, develop synergistic cardiac dysfunction, particularly when Trastuzumab is combined with doxorubicin [1], [2], [3]. A variety of transgenic mouse studies have extended our awareness of the role of ErbB2 in the heart. For example, ErbB2 knockout mice die in utero at E10.5 due to the defective cardiac trabeculation [4], [5], [6]. In conditional deletion models, adult mice develop heart failure [7], [8], and isolated cardiomyocytes from these mice are more sensitive to doxorubicin [7].

While the studies on ErbB2 in the heart have focused on consequences of blocking activity of this kinase, there is also clinical evidence that, in humans, ErbB2 is expressed at variable levels in cardiomyocytes. One of the most illuminating studies used SPECT imaging and identified differences in cardiac anti-ErbB2 binding to human hearts [9]. This research group originally planned to image the binding of radiolabeled anti-ErbB2 (Herceptin) to breast cancers but unexpectedly found that anti-ErbB2 also bound to the hearts of some patients. Since only the patients that showed anti-ErbB2 cardiac binding subsequently developed cardiac toxicity, it has been suggested that variable levels of ErbB2 expression among individuals might be an important determinant of susceptibility to doxorubicin and Herceptin toxicity.

In previous studies of cardiac toxicity of doxorubicin in the rat, we noted that doxorubicin treatment results in induction of ErbB2 expression [10]. While it appears that an up-regulation of ErbB2 in hearts in cancer patients might initially offer protection from toxic effects of doxorubicin, long-term effects of ErbB2 over-expression, particularly when not induced as a response to doxorubicin treatment, are unknown. We therefore generated two transgenic lines of mice with cardiomyocyte-specific ErbB2 over-expression to investigate consequences of long-term over-expression of ErbB2 in the heart.

## Methods

### Animals

This study was performed in strict accordance with the recommendations in the Guide for the Care and Use of Laboratory Animals of the National Institutes of Health. The protocol was approved by the Committee on the Ethics of Animal Experiments of the Johns Hopkins Medical Institutions (Animal Welfare Assurance # A-3273-01).

### Transgenic Constructs and Mouse Lines

Rat ErbB2 mRNA was isolated and converted to cDNA. The 5 kb cDNA fragment was then subcloned into the BamHI-SalI site of the cardiac specific expression vector, α-myosin heavy chain promoter construct (kindly provided by Dr. Jeffrey Robbins), followed by polyadenylation signal from human growth hormone (hGH), located downstream of the insert. The B6SJLF1/J strain was used for pronuclear microinjection of the received fragment and production of the transgenic mice by Johns Hopkins Transgenic Core Facility. Founder animals were identified by PCR and Southern blotting. Two founders were used to develop two transgenic lines. All the wild type and transgenic mice were housed under a 12 hours light-dark cycle with free access to food and water.

### Necropsy

Mice were euthanized and weighed, and tibia lengths measured. Hearts were excised, weighed, and sectioned at mid-papillary level. In selected mice, left ventricle, right ventricle and septum were snap frozen and saved for the further molecular studies. The basal to mid-papillary level of the heart was fixed in 10% formalin and paraffin-embedded for the histological evaluation. Five micron sections were stained with hematoxylin and eosin (H&E) for the morphological evaluation, with Masson’s trichrome for detection of fibrosis, or wheat germ agglutinin for cardiomyocyte morphology.

### Chest Radiography

Faxitron X-ray MX-20 Specimen Radiography system (Faxitron Bioptics, LLC, Tuscan, AZ) was used to perform chest radiography. The mice (8–10 weeks old, 2 mice per genotype) were euthanized and anterio-posterior radiography was immediately performed. Voltage and integration time were adjusted to visualize the heart shadow. Following the chest radiography, the mice were euthanised, skin and anterior portion of ribs with the sternum were cut and raised to open the thoracic cavity and expose the heart and lungs, and photos of the opened chest cavity with exposed heart and lungs were taken to match the gross pathology with radiograph.

### Real-time PCR and Primers Design

Total RNA was isolated from the hearts of the wild type and ErbB2 transgenic mice (8–10 weeks old, 3 mice per genotype) as described [10]. Hypertrophy molecular markers atrial natriuretic peptide ANP (NPPA) and β-myosin heavy chain (MYH) were evaluated by quantitative real-time reverse transcriptase-polymerase chain reaction (qRT-PCR). The primers are listed in Table S1. Peptidylprolylisomerase A (PPIA) was used for RNA normalization [11]. The quantitative RT-PCR results were calculated by delta-delta Ct method using the mean of the delta Ct value of wild type mice as a normalization factor.

### Immunoblotting

Both lines of mice were evaluated by Western blotting for protein expression and pathway activation. 8–10 week old male mice for each genotype were evaluated (n = 7); for lapatinib studies 3–6 animals per group were used. Frozen left ventricle (40 mg) was rapidly homogenized in 200–300 µL of RIPA buffer (Table S2), and standard gel electrophoresis and immunoblotting were performed. The antibodies used are listed in Table S3. After incubation in anti-rabbit, anti-mouse (1∶5000; GE Healthcare, Piscataway, NJ) or anti-rat (1∶5000, Cell Signaling Technology, Danvers, MA) horseradish peroxidase-linked secondary antibody, blots were exposed to chemiluminescent substrate (Pierce, Rockford, IL) and exposed to CL-Xposure film (Pierce, Rockford, IL). Levels of AKT protein were measured to normalize protein quantities across samples.

### Immunoprecipitation

Lysates were prepared from the hearts of wild type and ErbB2 transgenic mice (8–10 weeks old, 7 mice per genotype; for lapatinib experiments, 12.5 or 31 days old mice were used, 3–4 mice per group). Total protein quantification was performed prior to immunoprecipitation. Lysates were incubated with anti-ErbB2 or anti-EGFR antibody (3 µg of antibody per 1000 µg of total protein) at 4°C for 2 hours.

IgA beads were washed with ice cold lysis buffer (Table S2) and centrifuged (3000RPM, 1 min), buffer was aspirated, and beads were mixed with antibody-treated lysates and incubated at 4°C overnight. Subsequently, beads were washed 3 times and centrifuged (3000RPM, 1 min), the supernatant was aspirated, urea sample buffer (Table S2) was added to beads, and the suspension was heated at 95°C for 5 minutes, followed by centrifugation (3000RPM, 1 min). Supernatant was then loaded on SDS running gel, and the standard immunoblotting protocol was followed. The resulting membrane was probed with anti-phospho-tyrosine, anti-ErbB2 or EGFR antibodies.

### WGA Staining

Unstained sections of formalin-fixed, paraffin-embedded heart tissues were de-parafinized in xylene and rehydrated in decreasing concentrations of ethanol (100%, 95%, 70%). Antigen retrieval was performed with Dako S1700 Target Retrieval solution (Dako North America, Carpinteria, CA) in a steamer. Nonspecific background was quenched and active aldehyde was blocked by 10 minutes incubation of the slides in 1 mg/ml NaBH4 (Sigma-Aldrich, St. Louis, MO) in PBS. The slides were stained with wheat germ agglutinin conjugated to Alexa Fluor 488 (Life Technologies (Invitrogen), Grand Island, NY) overnight and coverslipped with DAPI-containing anti-fading media (Vectashield (Vector Technologies, Burlingame, CA)). The resulting sections were visualized with Nikon Eclipse E600 microscope and micrographs were obtained. 8–10 week old male mice for each genotype were evaluated (n = 5–6); for lapatinib study 31 days old animals were used (n = 3–4).

### Adult Cardiomyocytes Isolation and Measurements

Cardiomyocytes were isolated from hearts of 8–10 weeks old wild type and ErbB2 transgenic mice (3–4 mice per genotype). The hearts were quickly removed from the chest after euthanasia and the aorta was retroperfused at 100 cmH_2_O and 37°C for 3 min with a Ca_2+_−free bicarbonate-based buffer, gassed with 95% O2–5% CO_2_ (Table S2). Enzymatic digestion was initiated by addition of 0.9 mg/ml collagenase type 2 (Worthington Biochemical Co., 299 U/mg) and 0.05 mg/ml protease type XIV (Sigma Chemical Co.) to the perfusion solution (6–7 min). Dispersed myocytes were filtered through a 150 µm mesh and gently centrifuged at 500 rpm for 30 seconds. Longitudinal surface areas of isolated cardiomyocytes were visualized with Nikon Eclipse 90 microscope, measured and calculated from the digitized micrographs with NIS Elements 3.10 Imaging Software.

### Echocardiography

Trans-thoracic echocardiography was performed on conscious mice using Acuson Sequoia C256 ultrasound machine (Siemens Corps, Mountain View, CA) equipped with the 15 MHz linear array transducer. The mouse heart was imaged by two-dimensional and M-mode approaches using the parasternal short axis view at a sweep speed of 200 mm/sec. Measurements were acquired using the leading-edge method, according to the American Echocardiography Society guidelines [11]. Left ventricle wall thickness and left ventricle chamber dimensions were acquired during the end diastolic and end systolic phase including: inter-ventricular septum (IVSD), left ventricular posterior wall thickness (PWTED), left ventricular end diastolic dimension (LVEDD) and left ventricular end systolic dimension (LVESD). Three to five values for each measurement were acquired and averaged for evaluation. The LVEDD and LVESD were used to derive fractional shortening (FS) to measure left ventricular performance by the following equation: FS (%) = [(LVEDD – LVESD)/LVEDD]×100.

For quantifying left ventricular geometrical changes and phenotype, relative wall thickness (RWT) and left ventricular mass (LV mass) were measured, and the RWT ratio was used to define left ventricle wall thickness in proportion to left ventricle cavity size using the following equation: (RWT) = 2×PWTD/LVEDD. Left Ventricular mass (LV mass) was calculated with the following equation: LV mass (mg) = 1.055[(IVST + LVEDD + PWTD)3−(LVEDD)3], where 1.055 is the specific gravity of the cardiac muscle. 8–10 week old male mice for each genotype were evaluated (n = 15–18). Cardiac output (CO) was calculated using the following equations: CO  =  SV×HR; SV  =  VTI×CSA, where: SV  =  stroke volume; HR  =  heart rate; CSA  =  cross sectional area of aortic root; VTI  =  the velocity time integral. Four to five animals per group (wild type and ErbB2 transgenic mice, 8–10 weeks old) were used with 8–10 peaks measured for each VTI measurement per mouse.

### Blood Pressure

Blood pressure was obtained using tail-cuff plethysmography (CODA2, Kent Scientific, Torrington, CT, USA) as previously described [12]. Conscious mice were placed in a restrainer on a warming pad and allowed to rest inside the cage for 10–15 min before the measurements were taken. Mouse tails were placed in a tail cuff, which was inflated and released several times to allow the mouse to be accustomed to the procedure. Four to five animals per group (wild type and ErbB2 transgenic mice, 8–10 weeks old) were used.

### Electrocardiography (EKG)

Electrocardiography recordings were performed in 14 conscious mice per genotype (8–10 weeks old). Surface probes were inserted subcutaneously and EKG signal (Standard lead II) was obtained for five to ten seconds using a PowerLab data acquisition system (ML866) and Animal Bio Amp (ML136; AD Instruments, Colorado Springs, CO, USA). LabChart Pro 7.2 software (AD Instruments, Colorado Springs, CO, USA) was used for automated EKG tracing analysis.

### Isoproterenol Administration for EKG Studies

At eight weeks of age, male or female mice (3–4 animals per genotype) were anesthetized with intraperitoneal injection of ketamine HCl (90 mg/kg) and xylazine HCl solution (10 mg/kg). Anesthetized mice were placed in a supine position on a temperature controlled heating pad. Isoproterenol (0.1 mg/kg, 0.5 mg/kg or 100 mg/kg) was administered with an intraperitoneal injection to wild type and ErbB2 transgenic mice. Body temperature was monitored with rectal probe and maintained at 37–38°C. 5 minutes of EKG signal were recorded prior to the isoproterenol injection, followed by 30 minutes of recording after the injection.

### Lapatinib Treatment

Wild type or ErbB2 transgenic mice male and female mice (P4.5) were treated with oral lapatinib (160 mg/kg) of lapatinib PO daily, (n = 4–7/group). 24G-1″ Gavage needles (Braintree Scientific, Inc., Braintree, MA) were used. Lapatinib (LC Laboratories, Woburn, MA) suspension was made fresh before each treatment. Lapatinib was diluted with a buffer containing 0.5% carboxymethylcellulose, 1.8% sodium chloride, and 0.4% Tween-80in dH_2_O. Because a percentage of unchanged lapatinib is excreted with feces, we assigned entire separately caged litters to either vehicle or lapatinib group. Animals were treated daily for 8 days and euthanized 2 hours following final treatments. Hearts were excised, weighed, and sectioned, with the apical part immediately frozen for further molecular studies and the basal part with the atria fixed in 10% formalin for histopathology. Tails were saved for genotyping.

In a separate dosing experiment, 10.5 days old pups (n = 5–12) were treated daily with 100 mg/kg of lapatinib, following the same scheme. The pups were euthanized 21 days after the treatment initiation.

### Statistical Methods

GraphPad Prism software (GraphPad, La Jolla, CA) was used to perform statistical analysis.

After determining means and standard deviations, the Student unpaired T-test was used to determine significance of differences between groups, with a P-value of <0.05 accepted as a significant difference. For survival statistics Log-rank (Mantel-Cox) test was used.

## Results

### ErbB2 Over-expression Induces Concentric Cardiac Hypertrophy in Novel Transgenic Mouse Lines

**Figure 1:**
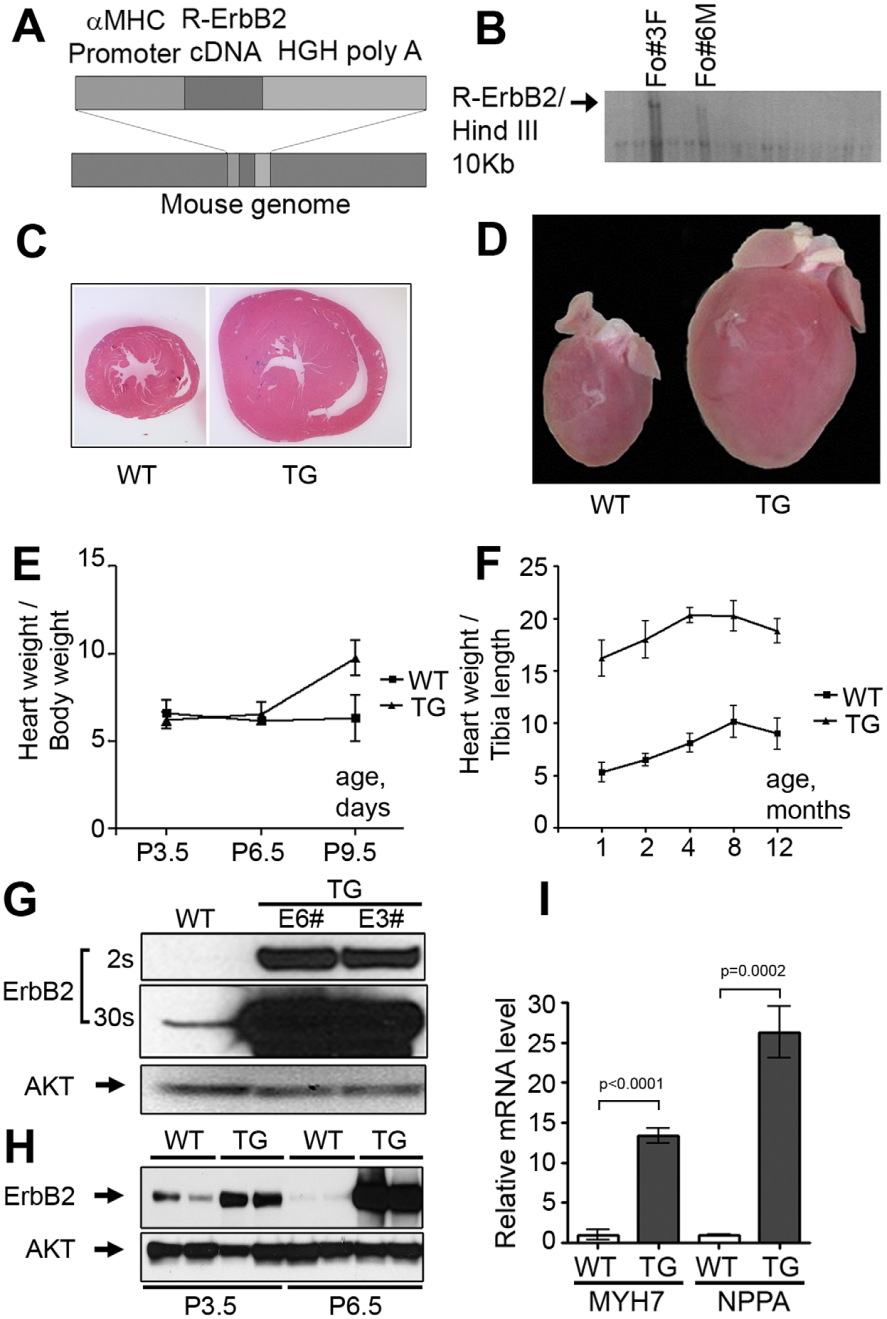
Concentric cardiac hypertrophy in ErbB2 transgenic mice. (A) Schematic illustration of DNA construct, consisting of cardiac-specific alpha-myosin heavy chain (αMHC) promoter, rat ErbB2 cDNA and polyadenylation signal from human growth hormone (HGH); (B) Identification of ErbB2 transgenic founder mice by southern blotting (tail DNA from 16 potential founders was used, two animals were found to have the transgene); (C) Hematoxylin-eosin staining (H&E) of transverse cross sections of the wild type and ErbB2 transgenic hearts. (D) Gross pathology of enlarged atria and ventricles in ErbB2 transgenic mice compared to wild type littermates. (E) Heart weight-to-body weight ratios of the wild type and ErbB2 transgenic mice at P3.5 (postnatal day 3.5), P6.5, P9.5 (n = 5–22 per genotype per age group). The data are presented as the mean ± SD. (F) Heart weight-to-tibia length ratios in adult mice. The transgenic-to-wild type heart weight ratio of 2.5–3 is maintained into adulthood (n = 4–27 per genotype per age group). The data are presented as the mean ± SD. (G) Western blot of ErbB2 protein in left ventricle protein extracts from wild type and ErbB2 transgenic mice. (H) Western blot of ErbB2 protein in left ventricle protein extracts from wild type and ErbB2 transgenic mice at P3.5 and P6.5 (n = 2 per genotype per age group). (I) m-MYH7 and m-NPPA expression was evaluated by quantitative RT-PCR in triplicates with mRNA isolated from the left ventricles of wild type and ErbB2 transgenic (n = 3 per genotype). The quantitative RT-PCR results were calculated by delta-delta Ct method using the mean of the delta Ct value of wild type mice as a normalization factor. The data are presented as the mean ± SD. The studies were performed on 8–10 weeks old mice, unless different age is specified.

Our laboratory developed two ErbB2-over-expressing transgenic (TG) mouse lines, using an ErbB2construct driven by the αMHC promoter for cardiac restricted expression [Figure 1a]. Two founder animals (#3 and #6) selected by screening of 16 mice [Figure 1b] were used to establish two separate breeding colonies. Heterozygotes have been continuously bred to maintain wild type controls and over-expressing littermates.

Body weights at birth were not different between the mice genotypes or ErbB2 mouse lines, and we noted no significant differences in survival rates of transgenic mice compared to wild-type littermates within the breeding colony over 20 months of observation (Figure S1). This data was obtained from colony management records and records of occasion rare deaths that occurred during ultrasound handling. We consider these mortality differences to be stress-related, with transgenic mice being more susceptible to adrenergic stress and associated mortality. In addition, we noticed increased mortality in older transgenic dams, which may be explained by multiple pregnancies-related cardiac remodeling, exaggerating the existing cardiac hypertrophy.

Transgenic mice from both lines (E6#, Figure 1c and d, Figure S2 and E3#, data not shown) exhibit marked cardiac hypertrophy as evident from gross pathology and radiographs. Heart weight-to-body weight ratios were comparable in transgenic and wild type mice up to postnatal day 6.5, with a significant 54% increase at P9.5 [Figure 1e]. Heart weight-to-tibia length ratio differences reached a maximum of about a 2.5-fold difference at 10–12 weeks of age [Figure S3b], after which slight, age-related parallel increases in the heart weight-to-tibia length ratio were observed in both transgenic and wild type mice [Figure 1f].

Protein levels of ErbB2in hearts of transgenic mice were measured by Western blot analysis [Figure 1g], and comparable levels of ErbB2 protein were observed in both lines (E3# and E6#). Lysates from the left ventricles of 8 weeks old transgenic mice and wild type littermates were compared. The ErbB2 protein levels were approximately 40 times higher in left ventricles of transgenic animals compared to wild type littermates, while ErbB2 levels in other organs were not changed (data not shown). There were no differences in protein expression between male and female mice or between E3# and E6# lines, thus E6# males were used for the majority of subsequent experiments.

We evaluated ErbB2 protein expression in wild type and ErbB2 transgenic mice at days P3.5 and P6.5 [Figure 1h]. Wild type mice have higher ErbB2 expression at P3.5 compared to wild type mice at P6.5 days. In ErbB2 transgenic mice due to the transgene promoter activation, ErbB2 expression dramatically increases between P3.5 and P6.5 days. At P3.5, heart weights are not different between wild type and ErbB2 transgenic mice, yet at P9.5 the difference becomes significant. At P3.5, ErbB2 protein expression in transgenic hearts is increased compared to wild type hearts before the hypertrophy becomes obvious, yet the relative ErbB2 protein expression between wild type and ErbB2 transgenic mice is much higher in P6.5 and in adult animals [Figure 1g, h] compared to P3.5 mice reflective of α-myosin heavy chain promoter transgene activation.

### ErbB2 Over-expression Causes Activation of Pro-hypertrophic Gene Program and Pro-survival and Translational Pathways

We confirmed by qRT-PCR that adult mice have increased levels of mRNA encoding for atrial natriuretic peptide(NPPA) and β-myosin heavy chain (MYH7), two characteristic mRNAs and proteins that increase in cardiac hypertrophy [Figure 1i].

**Figure 2:**
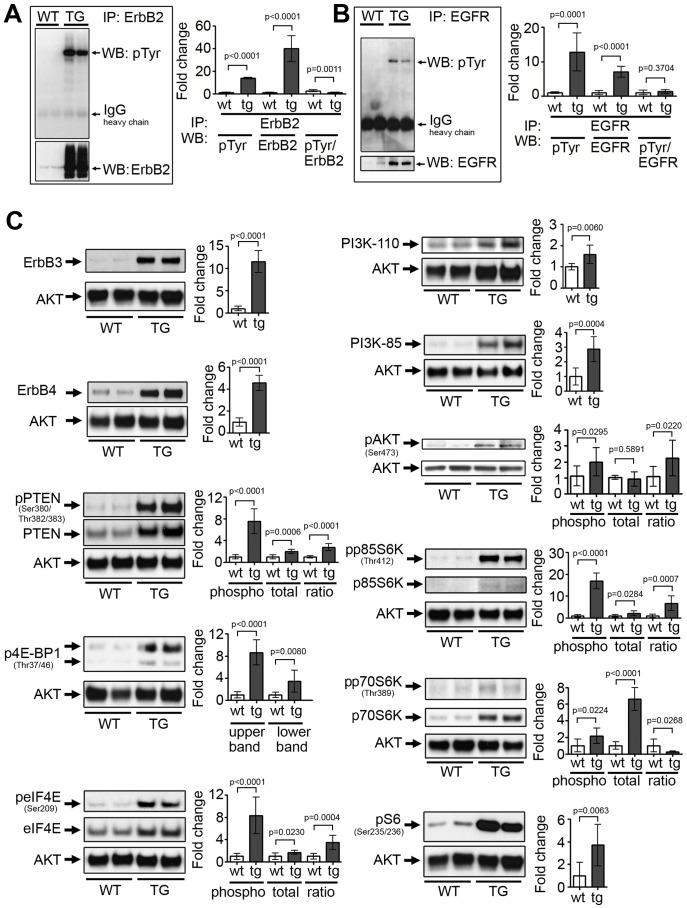
Up-regulation and activation of ErbB family proteins and downstream pro-hypertrophic proteins in the hearts of ErbB2 transgenic mice. (A) Total phosphorylation of ErbB2 and total ErbB2 levels (A), as well as total EGFR phosphorylation and total EGFR levels (B) were assessed by immunoprecipitation of ErbB2 or EGFR from the total left ventricle lysates and subsequent blotting with anti-phospho-tyrosine, anti-ErbB2 and anti-EGFR antibodies. Densitometry was performed using ImageJ software (n = 7 per genotype). The data are presented as the mean ± SD. (C) Representative western blots demonstrate activation of pro-survival, hypertrophic and translational signaling in the left ventricles of ErbB2 transgenic mice. Two bands of p4E-BP1 represent different levels of 4E-BP1 phosphorylation. AKT was used as loading control, since other proteins traditionally used as loading controls (GAPDH, tubulin, α-actin and β-actin) were variable in ErbB2 transgenic hearts. Densitometry was performed using ImageJ software (n = 7 per genotype). Phosphorylated proteins were normalized to the corresponding total proteins levels and AKT; phospho-protein-to-AKT and total protein-to-AKT ratios are also shown. All the studies were performed on 8–10 weeks old mice. The data are presented as the mean ± SD.

To explore signaling pathways affected by ErbB2 over-expression, we conducted a series of experiments to evaluate levels of proteins and levels of protein phosphorylation for key components of the ErbB2 pathway. Left ventricles from the two lines of ErbB2 transgenic mice showed highly similar patterns of protein expression or pathway activation. As expected, total phospho-ErbB2 is elevated in hearts of transgenic mice [Figure 2a], but surprisingly, ErbB2 over-expression induced a unique expression profile suggestive of a signal amplification effect. Specifically, we noted increased levels of proteins within the ErbB2 pathway that could be expected to facilitate and amplify pathway effects in the cell [Figure 2c]. ErbB2 over-expression activates and up-regulates cardiac pro-survival signaling and hypertrophic pathways, including PI3K/AKT pathway, a well-known pathway involved in increasing cardiomyocyte survival and protein translation during cardiac hypertrophy [13], [14], [15]. Both regulatory (p85) and catalytic (p110) PI3K subunits were markedly increased in hearts of ErbB2 transgenic mice. ErbB2 over-expression also induced a modest increase in AKT phosphorylation in transgenic hearts compared to wild type littermates, while total AKT levels remained unchanged. Total PTEN levels, as well as phospho-PTEN levels, were slightly increased in the ErbB2 transgenic hearts.

The EGFR family is represented in the heart by four proteins, EGFR, ErbB2, ErbB3 and ErbB4 receptor tyrosine kinases. We were surprised to see that total phospho-EGFR, and total EGFR, ErbB3 and ErbB4 levels were all elevated in ErbB2 transgenic hearts [Figure 2b,c].

We also explored signal transduction pathways that activate translation in ErbB2 transgenic hearts. Cardiac protein translation and cardiomyocyte hypertrophy is regulated through activation of p70S6K, subsequent S6 phosphorylation [16] and activation of translational regulators, such as eIF4E/4E-BP1 system [17]. Active p70S6K is required for hypertrophic transformation of neonatal rat cardiomyocytes in vitro [18], [19] and pressure overload-induced cardiac hypertrophy in rats in vivo [20]. These key proteins in the translational machinery were activated in ErbB2 transgenic hearts, as confirmed by increased phosphorylation of p70S6K, ribosomal S6 protein, eIF4E and 4E-BP1 [Figure 2c].

**Figure 3:**
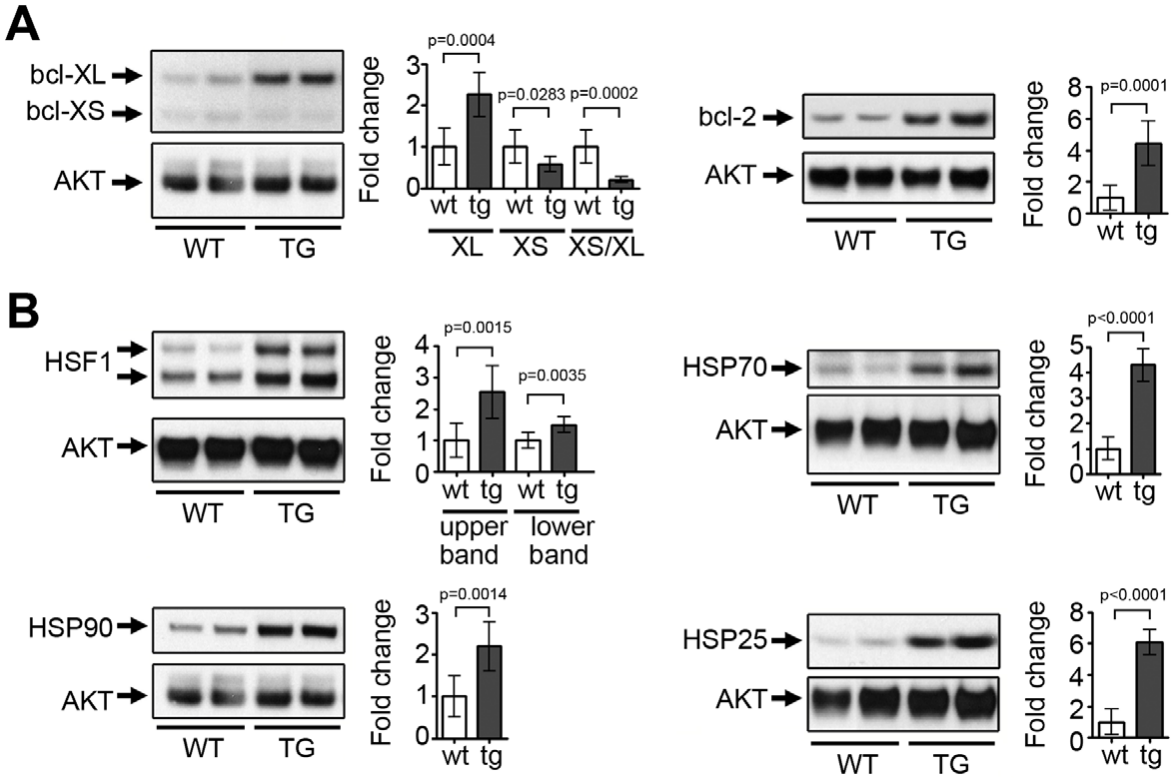
Up-regulation of pro-survival proteins and heat shock proteins in the hearts of ErbB2 transgenic mice. (A) Bcl-2 and Bcl-xL levels are increased, Bcl-xS levels are decreased and bcl-xS/XL ratio is reduced in left ventricles of ErbB2 transgenic mice. (B) Heat shock factor 1 (HSF1) and Heat shock proteins 25 (HSP25), 70 (HSP70) and 90 (HSP90) are elevated in left ventricles of ErbB2 transgenic mice. Two bands of HSF1 represent differentially phosphorylated HSF1 forms. Densitometry was performed using ImageJ software (n = 7 per genotype). All the studies were performed on 8–10 weeks old mice. The data are presented as the mean ± SD.

Next, we evaluated balance of pro- and anti-apoptotic Bcl-2 proteins. Bcl-2 family is comprised of anti-apoptotic (bcl-xL and bcl-2), and pro-apoptotic (bcl-xS, BAK, BAX) proteins. Correlation of cardiac ErbB2 expression and the balance of pro-survival bcl-xL and apoptotic bcl-xS proteins have been described [21], [22]. We found the same ratio pattern in hearts of ErbB2 transgenic mice, such as we noted a shift in the balance of bcl-x forms from predominantly the bcl-xS form (pro-apoptotic) to predominantly the bcl-xL form (anti-apoptotic) [Figure 3a], supporting the mechanism of ErbB2 in cardioprotection. In heart failure, the down-regulation of ErbB2 and ErbB4 receptors has also been correlated with decreased bcl-xL/xS ratios [23]. Additionally, we found that Bcl-2 levels were increased in ErbB2 transgenic hearts, supporting another mechanism of cardioprotection induced by ErbB2 [Figure 3a].

Another potential protective mechanism of ErbB2 over-expression in transgenic hearts, not previously associated with ErbB2 expression, is up-regulation of the transcription factor Heat shock factor 1 (HSF-1) and concomitant up-regulation of heat shock protein family, including HSP72, HSP25 and HSP90 [Figure 3b], all considered protective proteins. For example, HSP90 is a chaperone of both ErbB2 and EGFR and thus may be partially responsible for stability of these proteins in ErbB2 transgenic mice cardiac hypertrophy. HSF1 and its target HSP genes were shown to be induced in exercise-induced adaptive hypertrophy, but not pressure-overload maladaptive hypertrophy [24]. Supporting our finding in the heart, HSF1 protein levels and activation were shown to be induced by ErbB2 over-expression in a breast cancer cell line, while the mechanism of this ErbB2 connection to HSF1 is still unknown [25].

### ErbB2 Over-expression Induces Cardiomyocyte Hypertrophy with Myocardial Disarray

**Figure 4:**
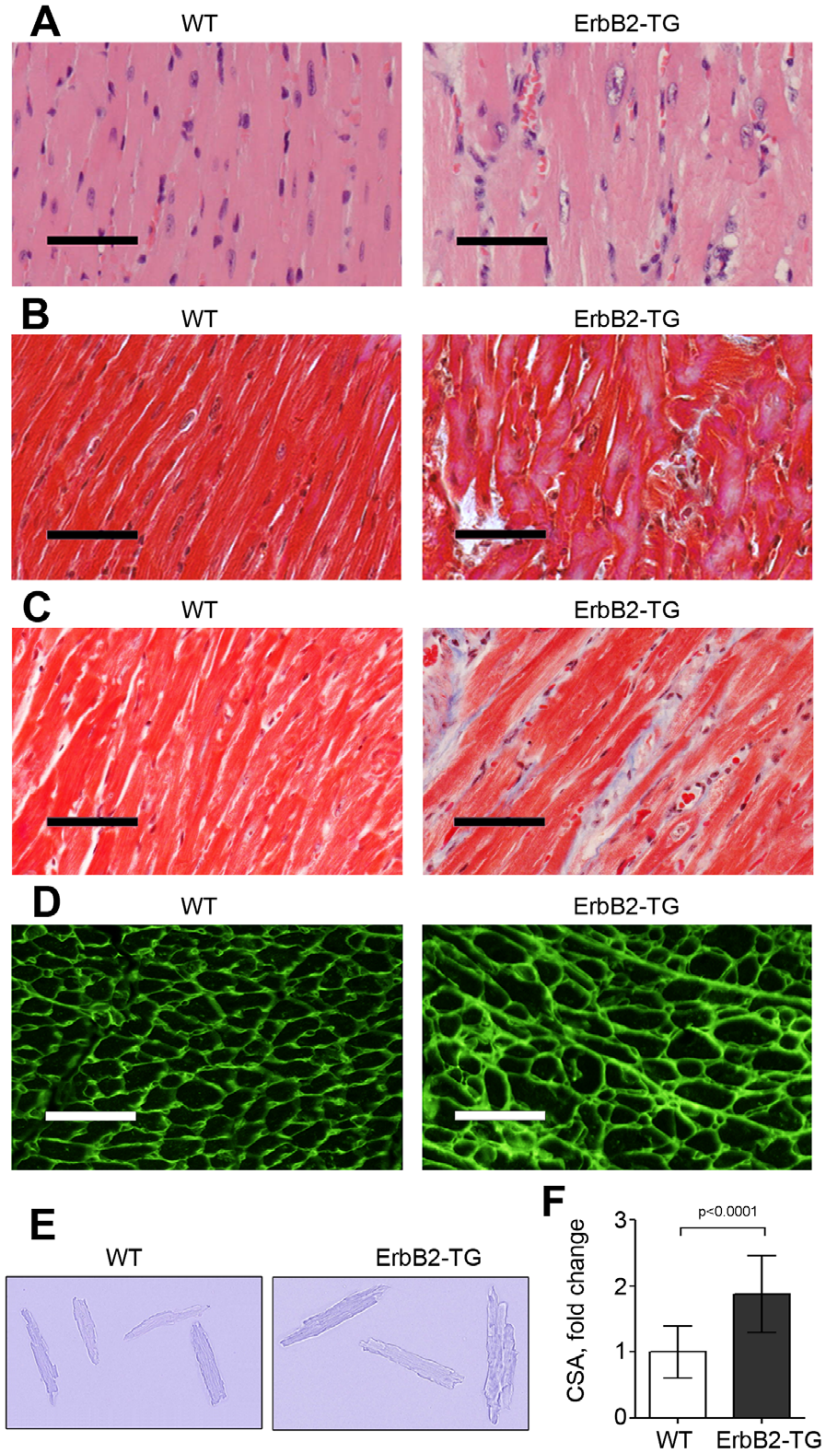
Cardiomyocyte hypertrophy and age-related interstitial fibrosis in ErbB2 transgenic mice. (A) H&E staining shows hypertrophied cardiomyocytes with enlarged nuclei in ErbB2 transgenic mice compared to wild type mice. Bar = 50 um. Masson trichrome staining was used to evaluate fibrosis in the hearts of 2 months (B) and 6 months old (C) wild type and ErbB2 transgenic mice. Bar = 50 um. (D) Representative images of WGA (wheat germ agglutinin) staining of left ventricle sections of wild type and ErbB2 transgenic mice hearts. (E) Isolated unstained cardiomyocytes from wild type and ErbB2 transgenic mice hearts. (F) Measurement of area of cardiomyocytes, isolated from wild type and ErbB2 transgenic hearts. 12–25 cardiomyocytes (3–4 mice per genotype) were used for calculations. The data are presented as the mean ± SD. All the studies were performed on 8–10 weeks old mice, unless different age is specified.

**Figure 5:**
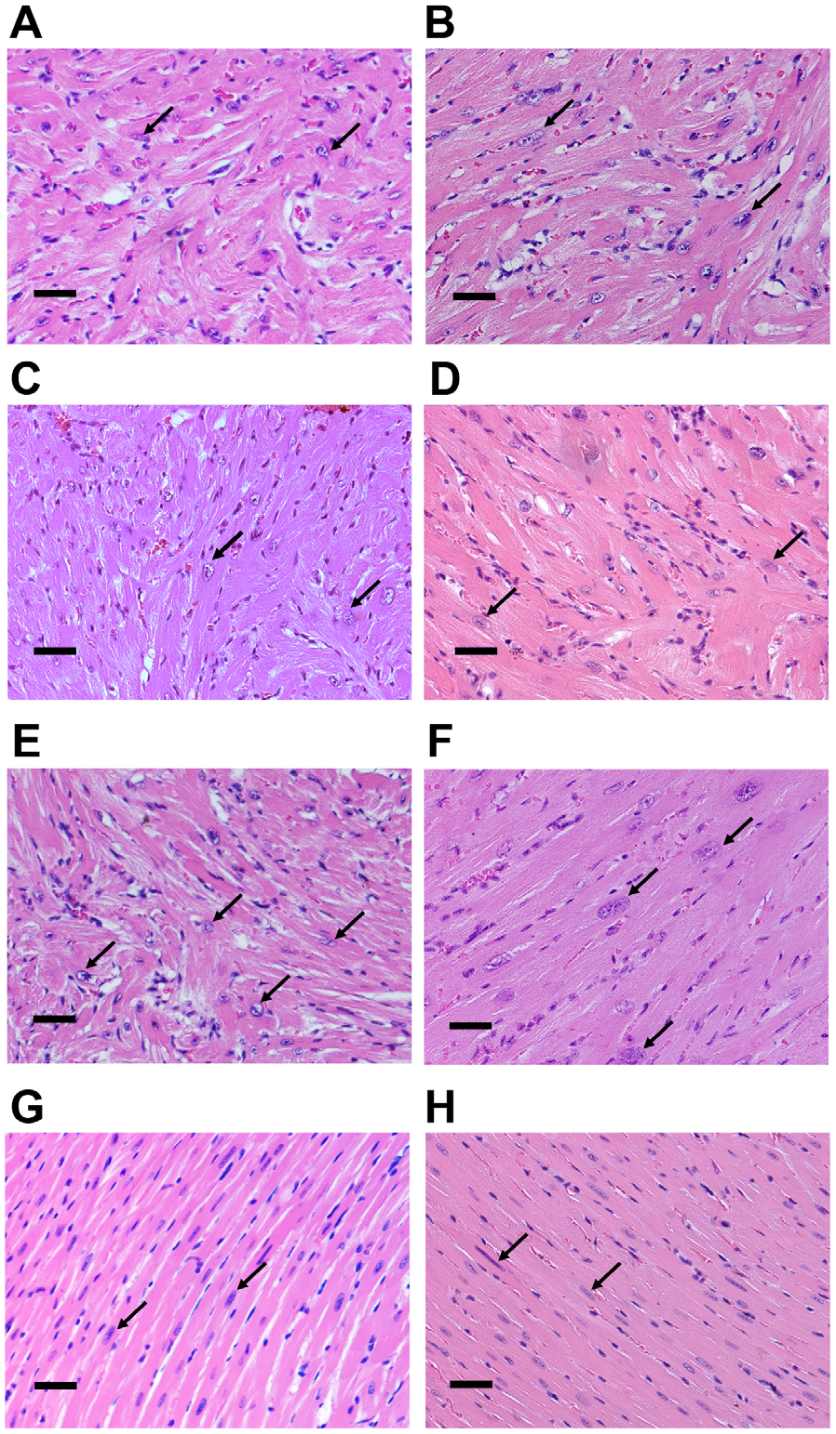
Histopathological evaluation of myocardial disarray in left ventricles and septa in ErbB2 transgenic mice. H&E staining of the heart sections of wild type and ErbB2 transgenic hearts. Left ventricles and septa of wild type and ErbB2 transgenic mice were evaluated for cardiomyocytes alignment and cell size. Enlarged nuclei are shown with arrows. Representative H&E stained sections from different heart regions are compared. Bar = 25 um. All the studies were performed on 8–10 weeks old mice. (A,C) ErbB2 transgenic mouse septum. (B,D,F) ErbB2 transgenic mouse left ventricle. (E) ErbB2 transgenic mouse junction of septum and left ventricle. (G) Wild type septum. (H) Wild type left ventricle.

Remarkably, ErbB2 over-expressing hearts have diffuse myocardial hypertrophy extending throughout the entire myocardium, with strikingly enlarged nuclei [Figure 4a, Figure 5a–f]. Masson’s Trichrome staining did not reveal obvious differences in fibrosis between transgenic and wild type mice at 8 weeks, but interstitial, perivascular and endocardial fibrosis did increase with age, as seen in the hearts of 6 months old transgenic mice compared to the age-matched littermates [Figure 4b and c]. Fibrosis continued to increase with age, as seen in 12 month- old ErbB2 transgenic mice compared to wild type mice [Figure S4]. Mineral deposits in fibrotic tissue were also observed in the left ventricular subendocardium in ErbB2 over-expressing hearts in mice as young as 4 weeks of age, without having deleterious effects on life span.

Microscopic studies confirmed that hypertrophy is related to increase in the size of individual cardiomyocytes. Cross sectional diameters of the cardiomyocytes were found to be increased in ErbB2 mice compared to the wild type mice, upon review of mid-papillary level transverse sections of hearts [Figure 4d]. In separate *in vitro* experiments, cardiomyocyte surface area was also measured in isolated cardiomyocytes; and ErbB2 over-expressing cardiomyocytes were found to be substantially larger than those of wild-type littermates, with a mean increase of surface area of 1.8 fold [Figure 4e and f].

A distinctive morphological feature of hypertrophic cardiomyocytes in ErbB2 transgenic mice is myocardial disarray, defined as disordered arrangement of cardiomyocytes with respect to one another. Disarray is more prominent in the septum but also was observed to a lesser degree throughout both ventricular walls of ErbB2 transgenic mice [Figure 5a–f] compared to wild type mice [Figure 5g, h]. The disarray in ErbB2 transgenic mice is characterized by a herring-bone, sometimes haphazard pattern of cardiomyocytes, with markedly enlarged cardiomyocytes and nuclei [black arrows] compared to the wild-type hearts [Figure 5a–h].

### ErbB2 Over-expression Induces Concentric Cardiac Hypertrophy, but does not Lead to Heart Failure

**Figure 6:**
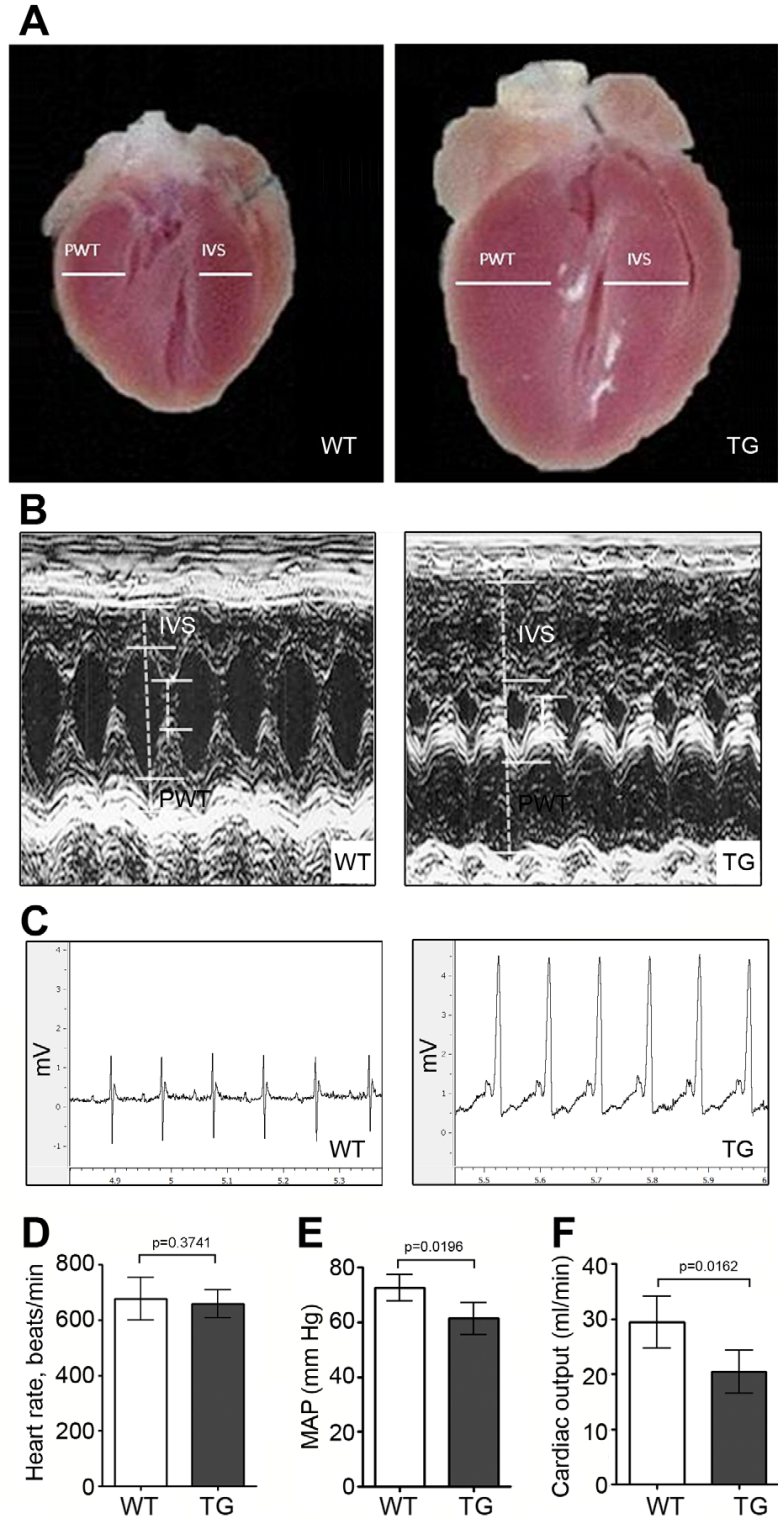
Gross pathology, echocardiography and electrocardiography of wild type and ErbB2 transgenic hearts. The wild type mice representative images are on the left and ErbB2 transgenic mice images are on the right of the panel. (A) Gross pathology reveals significant cardiac hypertrophy in ErbB2 transgenic mice. Longitudinal sections demonstrate hypertrophy of the heart walls and smaller left ventricle and right ventricle chambers in ErbB2 transgenic mouse. (B) M-mode produced by echocardiographic imaging demonstrates marked hypertrophy of the heart walls and reduction of left ventricle chamber in ErbB2 transgenic mice. IVS - interventricular septum, PWT – left ventricle posterior wall. 8–10 weeks old mice were used. (C) EKG was recorded in awake wild type and ErbB2 transgenic mice. Representative EKG tracings for each genotype are shown. (D) EKG was used for calculating the heart rate in wild type and ErbB2 transgenic mice (n = 14 per genotype). The data are presented as the mean ± SD. (E) Blood pressure was recorded in wild type and ErbB2 transgenic mice (n = 4–5 per genotype). The data are presented as the mean ± SD. MAP – mean arterial pressure. (F) Cardiac output was quantified in wild type and ErbB2 transgenic mice (n = 4–5 per genotype). The data are presented as the mean ± SD. All the studies were performed on 8–10 weeks old mice.

Cardiac hypertrophy can lead to a decrease of cardiac function with subsequent heart failure and death. We performed echocardiographic studies to compare functional performance and morphology in the transgenic mice and wild type littermates by examining male mice at 8 weeks of age (n = 15–18/group). Gross pathological specimens of longitudinal cross sections of heart demonstrate concentric hypertrophy in ErbB2 transgenic mice [Figure 6a]. Additionally, M-mode comparisons of echocardiograms highlight marked concentric cardiac hypertrophy.

Table 1 summarizes cardiac function and morphology. Comparing ErbB2 transgenic mice to wild type mice, we noted left ventricle wall thickness parameters (IVSD: 2.23±0.15 vs. 0.98±0.07 mm; PWTED: 2.24±0.13 vs. 0.91±0.08 mm; RWT: 2.12±0.34 vs. 0.63±0.1; and LV mass: 299.8±54.49 vs. 91.86±12.32 mg) consistent with the concentric hypertrophy observed at necropsy. The left ventricle cavity (LVEDD 2.16±0.33 vs. 2.91±0.28 mm) was significantly smaller during diastole in ErbB2 transgenic mice, suggesting concentric left ventricular hypertrophy as shown in representative M-mode [Figure 6b]. These changes are age-dependent, and at 2 months of age, the increase in LVESD (1.09±0.09 vs. 0.94±0.17 mm) and small decline in FS (56.31±2.35% vs. 62.51±2.41%) are still within the limits of normal function and morphology. Cardiac output and blood pressure were significantly reduced in transgenic mice, compared to wild type littermates [Figure 6e,f] but not to a degree which would be reflective of heart failure. Remarkably, the hypertrophy induced by ErbB2 over-expression does not progress to overt heart failure. At 12 months, FS is 61.20+/−2.63% versus 43.71+/−11.11% (wild type vs. transgenic). At 17–18 months, the fractional shortening in ErbB2 transgenic mice did not decline further and was maintained at 45.8+/−8.68% (n = 4).

**Table 1:** Cardiac function and morphology in wild type and ErbB2 transgenic mice.

Parameters	WT (n = 15)	TG (n = 18)	*t-test (p)*
LVEDD (mm)	2.91±0.28	2.16±0.33	0.0001***
LVESD (mm)	0.94±0.17	1.09±0.09	0.007**
IVSD (mm)	0.98±0.07	2.23±0.15	0.0001***
PWTED (mm)	0.91±0.08	2.24±0.13	0.0001***
FS (%)	62.51±2.41	56.31±2.35	0.0001***
LV mass (mg)	91.86±12.32	299.8±54.49	0.0001***
RWT	0.63±0.1	2.12±0.34	0.0001***

Echocardiographic parameters of ErbB2 transgenic mice confirm concentric hypertrophy consistent with necropsy results. Trans-thoracic echocardiography was performed on conscious 8–10 weeks old wild type (WT) and ErbB2 transgenic (TG) mice. Two-tailed t-test with **p<0.001, ***p<0.0001, Mean±SD.

### ErbB2 Transgenic Mice Possess Electrophysiological Features of Cardiac Hypertrophy

Cardiac hypertrophy in humans is characterized by a number of specific electrocardiographic features. To evaluate electrophysiological parameters of ErbB2 over-expressing hearts, we evaluated a total of 28 mice (14 per genotype), aged 8–10 weeks, by electrocardiography. Representative EKG tracings for each genotype are shown in [Figure 6c]. The EKG parameters of wild type and transgenic mice were compared. Heart rate was similar between wild type and ErbB2 transgenic mice [Figure 6d]. The P wave represents a depolarization of atria. P wave duration varied among transgenic mice, being increased in the most of them; biphasic, two-peaked and tall P waves were also common, corresponding to the left and right atria hypertrophy. PR interval was shortened in transgenic mice, which, although is not common, but may occur in cardiac hypertrophy. Other features, characteristic of cardiac hypertrophy, were also observed including significantly increased QRS complex voltage and duration, left axis deviation, and repolarization abnormalities (electrocardiographic strain pattern) [26], [27]. The distinctive differences observed between genotypes allowed EKGs to be routinely used to phenotype litters into wild type or ErbB2 genotypes for longitudinal *in vivo* experiments.

### Isoproterenol Induces Cardiac Arrhythmias and Death in ErbB2 Transgenic Mice

**Figure 7:**
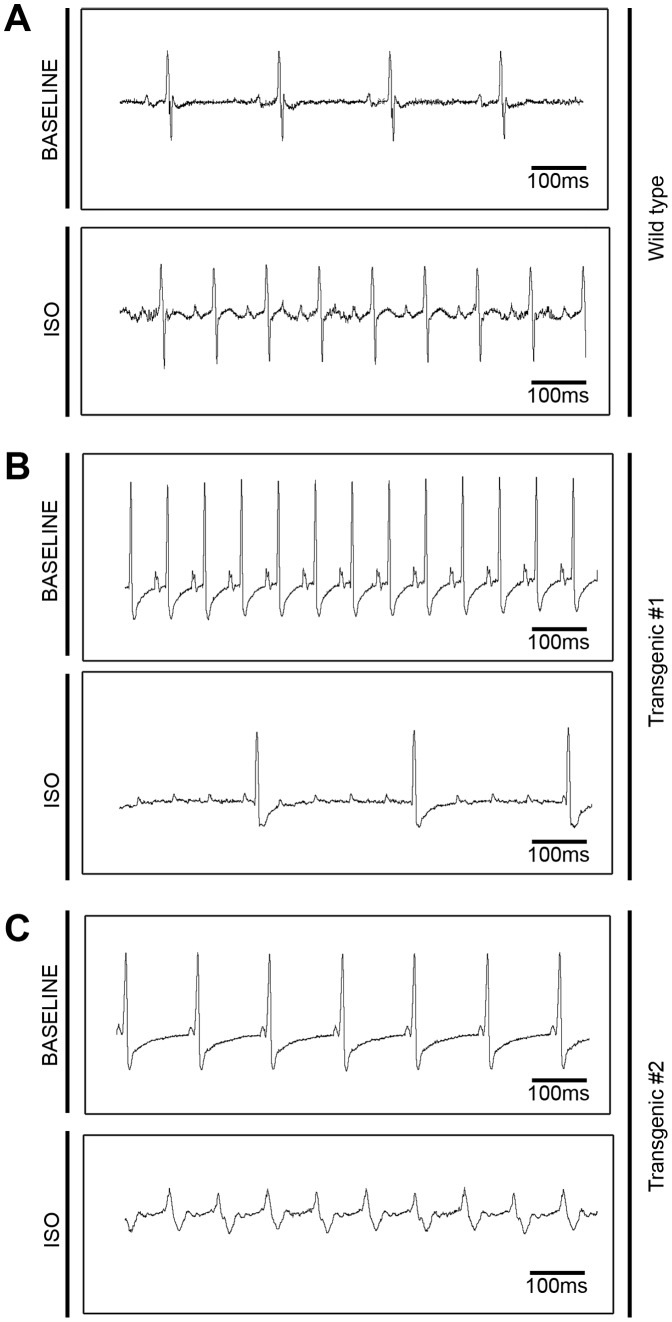
Isoproterenol treatment induces increased incidence of arrhythmias and sudden death in ErbB2 transgenic mice. Wild type (A) and ErbB2 transgenic (B) mice (n = 3–4 per genotype per treatment group) were anesthetized, and baseline EKG was recorded for 5 minutes. Isoproterenol (0.1 mg/kg, 0.5 mg/kg or 100 mg/kg) was injected intraperitoneally and EKG was recorded for 30 minutes. EKG tracings from 2 different ErbB2 transgenic mice are displayed to present 2 different types of arrhythmias. All the studies were performed on 8–10 weeks old mice.

Although ErbB2 transgenic mice show cardiac hypertrophy-specific EKG changes, in general we did not note any arrhythmias during routine EKG recording. However, after recognizing the sudden death of several transgenic animals with routine handling, we further explored a possible increased tendency for transgenic animals to develop arrhythmias with adrenergic stimulation. Under ketamine and xylazine anesthesia, wild-type and ErbB2 transgenic mice responded to the standard dose of isoproterenol (0.1 mg/kg, 0.5 mg/kg or 100 mg/kg) administration with comparable increases in heart rate, at 6–9 seconds after the injection. However, ErbB2 transgenic mice developed progressive electrocardiographic changes, including decreased R wave amplitude, widened QRS complex, and complex arrhythmias, followed by asystole and eventually death 5 to 8 minutes after isoproterenol administration. These arrhythmias included atrio-ventricular blocks, and in some cases ventricular tachycardia [Figure 7b,c]. In ErbB2 transgenic mice, 100% mortality was observed with isoproterenol with dosages as low as 0.1 mg/kg, 1/1000of the standard dosage (100 mg/kg) routinely tolerated by wild type littermates. Wild-type littermates maintained increased heart rate until the end of the 30-minute period of recording [Figure 7a]; in some mice, heart rate slowed, followed by sinus bradycardia, but heart rates in all wild type animals normalized with time.

In a separate experiment, 4–5 conscious mice per genotype were injected with 0.1 mg/kg isoproterenol, and in this setting, transgenic mice had fewer arrhythmias than the group of mice that were anesthetized. However, when these mice were returned to cages for monitoring, 100% of ErbB2 transgenic mice died, usually within several hours, after isoproterenol injection.

### Lapatinib Treatment Reduces both Cardiac Hypertrophy and ErbB2activation

Next, we used lapatinib, a pharmacological inhibitor of ErbB2 phosphorylation to induce pathway inactivation and determine whether cardiac hypertrophy in ErbB2 transgenic mice is dependent on translation pathway activation. Lapatinib is a small molecule reversible inhibitor of EGFR (IC_50_ = 11 nM) and ErbB2 (IC_50_ = 9 nM) tyrosine kinases. We chose lapatinib in our studies because it is commonly used in cancer patients and evaluating any potential toxicity would be also beneficial to uncover. Unfortunately, there were no substitute compounds that are specific inhibitors of only ErbB2. Lapatinib binds to cytoplasmic ATP-binding site of the ErbB2 and EGFR and blocks its phosphorylation and activation, with subsequent inhibition of downstream pathways. Lapatinib peak plasma levels occur 3–6 hours (in humans) [28], 1–2 hours (in mice) [29], [30] after oral administration.

We hypothesized that ErbB pathway inhibition would block or blunt cardiac hypertrophy initiation in mice with constitutive over-expression of ErbB2. We performed two separate experiments, varying the dose and the age when the study began. In one study, we began lapatinib treatment at P4.5 and in another study we began treatment at P10.5.

**Figure 8:**
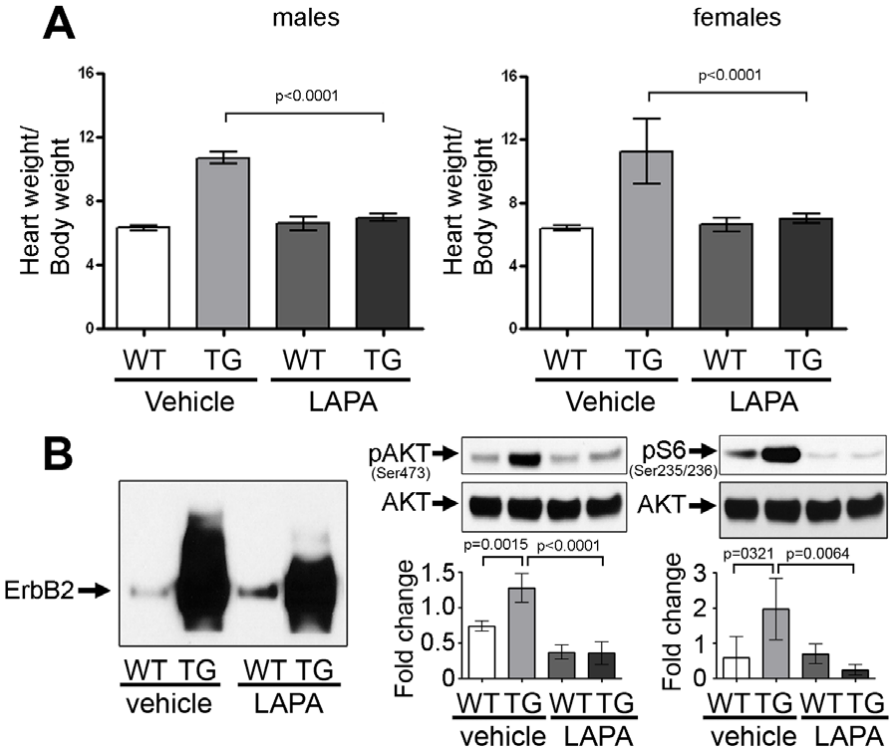
Lapatinib blocks hypertrophy development in ErbB2 transgenic mice. P4.5 male and female mice were treated with lapatinib via gavage for 8 days until P12.5 (n = 4–7 per genotype per treatment group per sex). (A) Increased heart weight to body weight ratio in ErbB2 transgenic mice at P12.5. Lapatinib treatment significantly reduces this ratio both in males and females. The data are presented as the mean ± SD. (B) Representative western blots of total ErbB2, phospho-AKT and phospho-S6 proteins in wild type and ErbB2 transgenic mice, treated with vehicle or lapatinib. Densitometry was performed using ImageJ (n = 4–6 per genotype per treatment group per sex). The data are presented as the mean ± SD.

In general, the molecular findings were similar in the two lapatinib dosing schemes, although starting the treatment before significant transgene activation and hypertrophy onset, induces a more substantial result in heart weight to body weight reduction in the younger group treated. In the 4–5 day old mice, treatment was given orally by gavage with 160 mg/kg of lapatinib for 8 days to test whether lapatinib can prevent the development of cardiac hypertrophy before it commences. Lapatinib administration successfully prevented hypertrophy development in ErbB2 transgenic mice, both in males (p<0.0001) and females (p<0.0001) while not affecting wild type littermates [Figure 8a]. Lapatinib treatment also reduces ErbB2 protein expression and significantly reduces levels of pAKT, and pS6 compared to wild type littermates resulting in abolishment of activation of ErbB2-dependent pathways and hypertrophic phenotype [Figure 8b]. A pathologist blinded to the treatment and genotypes reviewed the slides from this study. There was no evidence of cell death in the vehicle or lapatinib treated transgenic or wild type mice seen by histopathology or in TUNEL staining (data not shown).

#### Lapatinib also reduced the heart weights when given to older ErbB2 transgenic mice

Ten day old transgenic mice already have significant cardiac hypertrophy, which can be detected morphologically, on the necropsy, or electrophysiologically by EKG. At P9.5 the transgenic hearts are on average 1.5 times larger than the wild type hearts [Figure 1 and Figure S3a]. We hypothesized that lapatinib may delay further increase in cardiac mass in older ErbB2 transgenic mice. The mice were treated orally by gavage with lapatinib at 100 mg/kg, starting at P10.5 for 21 days. A lower dose was used in this study due to the extended period of dosing. Additionally, this dose is the most common dose used in the literature for chronic cancer studies.

**Figure 9:**
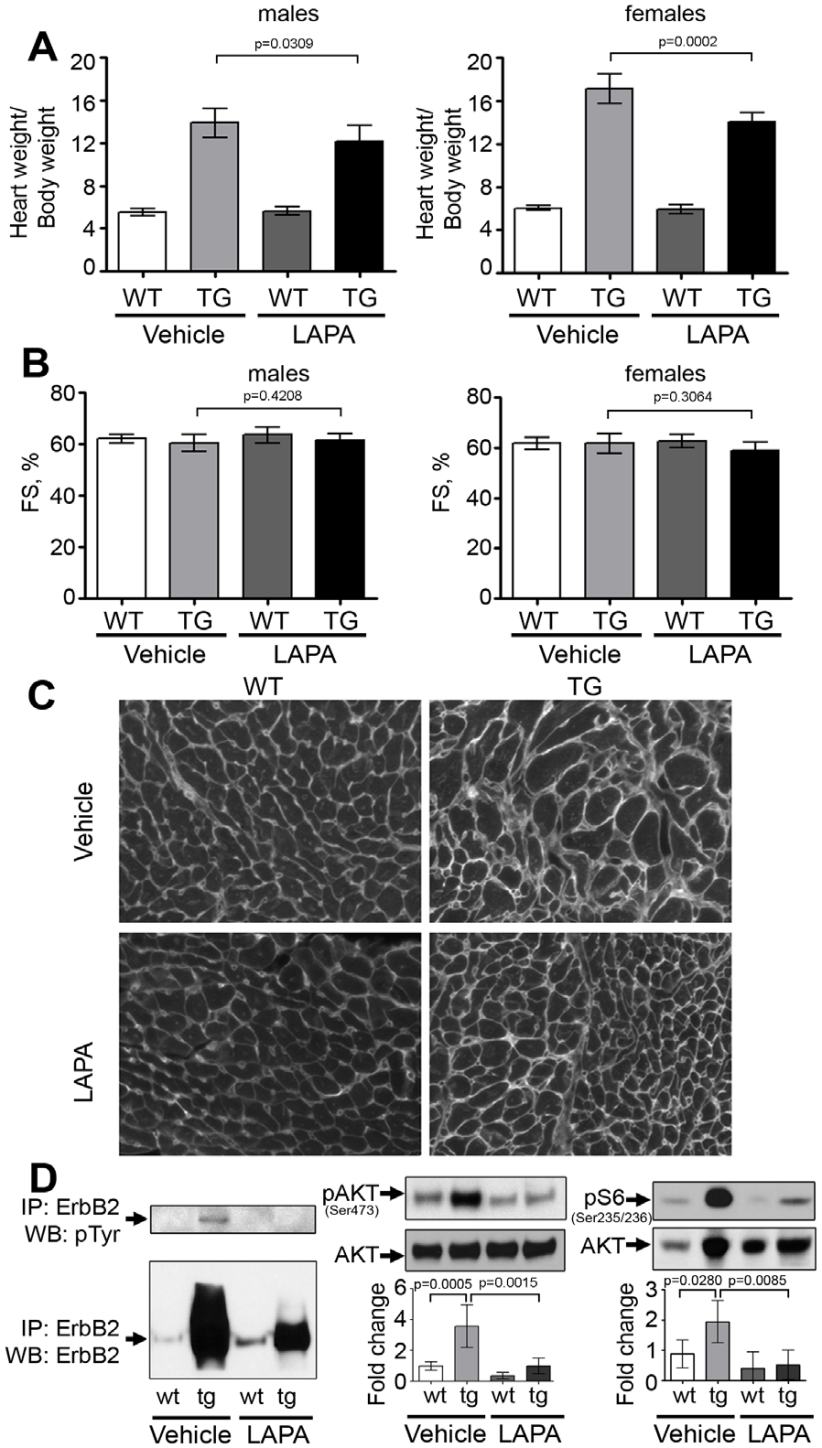
Lapatinib reduces hypertrophy in ErbB2 transgenic mice. Wild type and ErbB2 transgenic male and female mice were treated with lapatinib via gavage for 3 weeks, starting at P10.5 (n = 5–12 per genotype per treatment group per sex). (A) Increased heart weight to body weight ratio in ErbB2 transgenic mice compared to wild type mice, which is reduced with lapatinib treatment (n = 5–12 per genotype per treatment group per sex). The data are presented as the mean ± SD. (B) Fractional shortening was evaluated in wild type and ErbB2 transgenic mice, treated with vehicle or lapatinib (n = 5–12 per genotype per treatment group per sex). The data are presented as the mean ± SD. (C) WGA staining reveals cell size variation in the hearts of ErbB2 transgenic mice, compared to wild type littermates, including large, medium and small-sized cells. Lapatinib reduces the size of cardiomyocytes in the ErbB2 hypertrophied cells. (n = 3–4 per genotype per treatment group). (D) Total ErbB2 phosphorylation was evaluated by immunoprecipitation in wild type and ErbB2 transgenic mice, treated with vehicle or lapatinib. Densitometry was performed using ImageJ software (n = 3–6 per genotype per treatment group). The data are presented as the mean ± SD.

We were able to perform immunoprecipitation and echocardiography studies in the older mice study since the hearts were large enough in 31 day old mice at euthanasia versus at 12 days old mice. ErbB2 cytosolic domain contains multiple sites of phosphorylation and partner factors binding sites. For complete estimate of ErbB2 activity, it is important to evaluate the total phosphorylation status of ErbB2, rather than phosphorylation of individual tyrosine residues. For this purpose, we performed immunoprecipitation of ErbB2 and the precipitate was probed for the total phospho-tyrosine. Mice were euthanized 2 hours after the lapatinib dose to evaluate ErbB2 status by immunoprecipitation and phospho-tyrosine immunoblotting. In vehicle treated group, transgenic mice had significantly higher phospho-tyrosine levels than wild types. Lapatinib administration decreased total ErbB2 phosphorylation in transgenic mice. In wild type mice the pTyr signal of ErbB2 was too low to detect with vehicle or lapatinib treatment. Surprisingly, total ErbB2 level in ErbB2 transgenic mice was decreased by lapatinib (occurred in both dosing models). A significant reduction in heart-to-body weight ratio in both genders of transgenic mice was observed (p = 0.0309 in males, and 0.0002 in females) [Figure 9a]. The heart function, assessed by echocardiography, was not altered significantly in either wild type or transgenic mice after lapatinib treatment [Figure 9b]. In parallel with the modest but significant reduction of HW/BW, phosphorylation of AKT and pS6 was reduced [Figure 9d], similar to the other dosing model (figure 8b).

Lapatinib treatment only reduced the HW/BW in the mice with ErbB2 over-expression, not the wild type mice. Correspondingly, the extent of hypertrophy and numbers of hypertrophic cells were reduced by lapatinib treatment in transgenic hearts [figure 9c]. In general, ErbB2 over-expression induces an extensive variation of cardiomyocyte size with a mixture of hypertrophic cells, normal-sized cardiomyocytes, and even some unusually small cardiomyocytes. Lapatinib reduced the number of large cells in the ErbB2 transgenic mice and did not appear to have an effect on cardiomyocyte sizes in control littermate’s hearts. There was no evidence of cell death in the vehicle or lapatinib treated mice. TUNEL staining revealed no evidence of cell death in this experiment (data not shown), supporting our echocardiography studies that demonstrated normal function in mice treated with lapatinib for 21 days.

## Discussion

ErbB2 over-expression in the mouse heart leads to concentric hypertrophy with significant increase in sizes of individual cardiomyocytes. Remarkably, animals with ErbB2-induced cardiac hypertrophy do not develop heart failure. But mice with hypertrophic hearts are susceptible to arrhythmias, which are readily triggered by isoproterenol, and occasionally experience sudden death caused by routine handling of animals. ErbB2 over-expression activates cardiac pro-survival signaling and hypertrophic pathways in cardiomyocytes, including the PI3K/AKT pathway, which regulates cardiomyocyte survival and protein translation. ErbB2 over-expression also leads to up-regulation of the pro-survival bcl-2 family of proteins in the heart, with an anti-apoptotic shift in the balance of pro-survival bcl-xL and apoptotic bcl-xS proteins. Thus, the hypertrophic effects of ErbB2 are likely related to the role of this protein as a key regulator of protein translation and the balance between survival and apoptosis of cardiomyocytes.

By defining a role for ErbB2 in inducing cardiac hypertrophy, our results reveal new insights into previously recognized phenomena in human heart patients. In humans, ErbB2 protein was shown to increase early in the development of the heart failure from multiple etiologies, while ErbB2 protein decreases in patients with terminal heart failure [23]. Previous studies and the work we present here, suggest that, although ErbB2 may drive hypertrophy in an attempt to improve heart function, ErbB2 expression is not maintained and heart failure ensues. Mechanisms that induce ErbB2 expression in the human heart are not currently known, but clues to these mechanisms could possibly be revealed by investigating pathways activated after unloading the heart subsequent to left ventricular assist device (LVAD) implantation. In limited investigations into these pathways, ErbB2 mRNA was found to be increased following unloading in 36 patients with severe heart failure (NYHA class IV) due to ischemic and non-ischemic cardiomyopathy [31], but ErbB2 protein levels were not evaluated in this study. Other investigators found that ErbB2 protein is shed into serum when patients are in heart failure and this correlates inversely with left ventricular ejection fraction [32], providing additional circumstantial evidence that ErbB2 protein and its signaling could be increased at some phases of development of human heart failure.

Clues to the down-regulation of ErbB2 in terminal heart failure may be related to mechanisms involving miRNA. For example, an inverse relationship of ErbB2 and miR7 was found in humans with dilated cardiomyopathy [33] in a setting where ErbB2 appears to have an important role in cardiomyocytes of humans approaching heart failure. It is possible that, in this situation, stressed cardiomyocytes transiently develop hypertrophy, driven by ErbB2 expression and activity. Based on human evidence noted above, induction (and then loss) of ErbB2 expression and signaling is likely important in the transition from hypertrophy to heart failure. While molecular inducers and molecular inhibitors of ErbB2 are not well understood, our animal model unequivocally for the first time establishes that activation of ErbB2 induces cardiac hypertrophy.

Decline in ErbB2 protein expression temporally correlating with transition to heart failure is also seen in animal models. In various experimental models of heart failure in animals, ErbB2 pathway is activated in early stages of heart failure but subsequently becomes inactivated in later stages of heart failure. For example, ErbB2 is up-regulated initially following doxorubicin induced damage before there is a systolic dysfunction in rats [10]. Yet in later stages of doxorubicin toxicity, in a mouse model, miR146 is responsible for reducing ErbB pathway activation by decreasing cardiac ErbB4 protein levels, hence decreasing protective signaling [34]. In a canine cardiac stress model, ventricular pacing induces ErbB2 phosphorylation, apparently (and surprisingly) without activation of AKT or ERK pathways [35]. Lastly, in an aortic stenosis model of hypertrophy in rats, with progression to heart failure, ErbB2 levels are maintained in hypertrophic hearts but decrease as heart failure develops [36], suggesting that ErbB2 pathway activity is important in preventing heart failure progression under conditions of stress. We recognize that in many situations of cardiac stress, up-regulation of ErbB2 as a protective response might occur only in the most stressed cells requiring a hypertrophic response, and not uniformly among all cardiomyocytes. If so, a significant up-regulation in ErbB2 protein may not always be appreciated by Western blotting techniques.

Since other models of hypertrophy lead to heart failure, our finding of a lack of heart failure in the ErbB2 mouse model is particularly remarkable. The propensity to cause hypertrophy without heart failure is also seen with over-expression of insulin-like growth factor 1 (IGF-1) [37] and insulin-like growth factor 1 receptor (IGF1R) [38]. Both ErbB2 and IGF1R share downstream proteins that are pro-survival in nature, explaining why heart failure is not seen with over-expression of these proteins. In another example, PI3K over-expression in the heart induces hypertrophy without heart failure [14], [39]. Multiple cardiac AKT over-expression models have been described, with various degrees of cardiac hypertrophy, which resulted in heart failure in some models, but not in others [13], [15], [40], [41]. A few factors can modulate the phenotypes between different AKT over-expression models, including fold increase in the transgene expression and different signaling pathways activated by constitutive versus inducible AKT over-expression. Concerning the comparison of AKT- and ErbB2-over-expressing mice, activation of survival pathways by ErbB2 aside of PI3K/AKT pathway, such as HSPs and bcl-2 family proteins, may contribute to a sustained survival of ErbB2 transgenic mice versus AKT transgenic mice with resulting heart failure. Also, increased total AKT levels in AKT over-expression models may provide additional signaling changes, while in ErbB2 over-expressing model AKT phosphorylation was modestly increased, without an increase in total AKT.

ErbB4 is another protein in the ErbB family that has been over-expressed experimentally in the heart, but surprisingly this over-expression does not result in hypertrophy. Tidcombe and colleagues over-expressed human ErbB4 in hearts of erbB4 knock-out mice to determine whether this over-expression could rescue hearts from the trabeculation defect seen in erbB4 complete knock outs [42]. While this over-expression of ErbB4 did rescue the trabeculation defect phenotype, neither the degree of erbB4 over-expression level, nor the hypertrophic phenotype, was reported in these mice. Phenotypic differences between our model and the erbB4 over-expression model may be explained by comparing signaling differences between these two mouse models, since multiple ligand and receptor combinations [43] could offer a plethora of cellular phenotypes [44], [45], [46]. Notably, each receptor tyrosine kinase possesses a set of -phosphorylation sites, activation of which may also define unique downstream activation patterns [47] As an example, NRG-induced ErbB2 heterodimerization with ErbB4 can activate Stat5, but both ErbB2-ErbB2 and ErbB4-ErbB4 homodimers lack this ability [48]. Differential activation of pro-hypertrophic versus proliferative pathways may explain why ErbB4 over-expression led to a proliferative response in cardiac myocytes, but not to hypertrophy [49].

**Figure 10:**
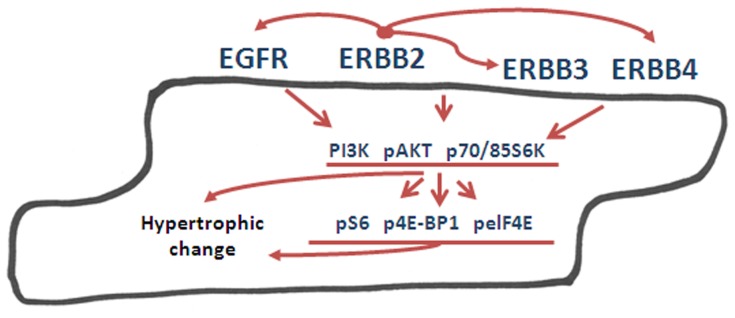
Schematic representation of the pathways involved in cardiac hypertrophy in ErbB2 transgenic mice. ErbB2 over-expression results in increased levels of other ErbB family members, EGFR, ErbB3 and ErbB4. ErbB family signals to downstream PI3K, AKT and p70/85S6K proteins, which leads to activation of translational machinery and hypertrophic phenotype.

A far as we can determine, this study is the first to demonstrate that ErbB2 over-expression regulates the expression of EGFR, ErbB3 and ErbB4, as well as both PI3K subunits. These findings may have relevance, not only to cardiac studies, but also for understanding the role of ErbB2 role in cancer biology. We suggest that ErbB2 is a major regulator of ErbB receptors family and its downstream signaling [Figure 10], and it is possible that ErbB2 controls a subset of proteins that drive cardiac hypertrophy either through receptor signaling or through ErbB2 effects in the nucleus, where ErbB2 translocates in cleaved [50] or intact [51] forms and has been linked to the trans-activation of the COX-2 promoter [51].

Comparing animal models with cardiac expression of signaling proteins, ErbB2 over-expression induces myocardial disarray, while PI3K, AKT and IGF1R over-expression do not induce myocardial disarray. Additionally, compared to Hypertrophic Cardiomyopathy mouse models [52], [53], [54], ErbB2 transgenic mice have a much greater degree of disarray, and thus may be useful in dissecting molecular mechanisms of disarray and the effects of myocardial disarray on cardiomyocyte physiology. Notably, myocardial disarray is commonly seen in human heart disease, including HCM, and it is linked to arrhythmias, syncope and sudden death [55], [56]. Currently, there is controversy on whether myocardial disarray induces arrhythmias. In some experiments, changes in myofiber direction were shown to interfere with conduction [57], [58], and the presence of disarray is a risk factor for arrhythmias development in some animal models [59]. Conversely, other studies show that occurrence of cardiac arrhythmias is not related to amount and distribution of the disarray [60]. In humans, a direct association between the disarray and sudden death has been accepted [61], [62], with ventricular tachycardia and fibrillation considered to be primary causes of sudden cardiac death in HCM patients. Defining molecular determinants of cardiomyocyte interaction with its cellular environment, including cells and extracellular matrix, in the ErbB2 transgenic mouse may uncover new insights into myocardial disarray and mechanisms of cardiac arrhythmias. Thus, our finding that ErbB2 expression in the heart induces both myocardial disarray and arrhythmias is potentially important for understanding the relationship between these phenomena in human disease.

Support for the use of this model for investigating the role of myocardial disarray in cardiac rhythm disturbances come from our findings that hearts of ErbB2 transgenic mice share distinctive electrophysiological features with human HCM patients, including increased QRS voltage and duration, ST segment and T wave abnormalities, as well as shortened PQ interval. Shortened PQ interval is observed in some patients with HCM, particularly in those without contractile proteins mutations, such as PRKAG2 mutations [63], [64], Danon disease [65], [66], Pompe disease [67] and Fabry disease [68], [69]. The electrophysiological disorder of ErbB2 transgenic mice heart physiology was also reflected in a particularly high sensitivity to the non-specific beta-adrenergic agonist, isoproterenol. Transgenic mice treated with modest doses of isoproterenol developed electrocardiographic changes and died shortly after isoproterenol injection, similar to the response seen in other HCM mouse models [70], [71]. This is consistent with humans with HCM and their heightened sensitivity to adrenergic stimulation and arrhythmias.

We propose that this novel model of ErbB2 over-expression in cardiomyocytes meets the criteria for the model of hypertrophic cardiomyopathy (HCM), which is characterized by cardiomyocyte hypertrophy, myocardial disarray and interstitial fibrosis [56]. Most of the HCM cases that have been genetically evaluated have mutated sarcomeric contractile proteins, but not all are associated with sarcomeric proteins (reviewed here) [55]. In humans with HCM, cardiac hypertrophy of a similar degree (as erbB2 transgenic mice) is not observed. Yet the similarities in the disease course, histopathology and functional changes between our model and human HCM allowed us to suggest our model as a potential model for human HCM, and particularly HCM not induced by contractile proteins mutations. Our model has distinctive cardiomyocyte disarray and chamber restriction/constriction consistent with HCM. Interstitial, subendocardial and perivascular fibrosis is minimal in 2 month old mice but does increase with age contributing to the stiffness of the heart, a feature shared with other mouse models and human HCM cases. Reduced cardiac output and decreased blood pressure, seen in ErbB2 transgenic mice, are other features often observed in HCM human patients particularly during exercise tests [72] and hypertrophic cardiomyopathy mouse models [73], [74].

The lapatinib studies were initiated to confirm the role of ErbB2 in cardiac hypertrophy. We assessed whether pathway activation, hypertrophy and heart weight/body weight would be affected by lapatinib or the age during treatment. We treated 2 groups of mice, one from 4.5 to 12.5 days of age, and another from 10.5 to 31 days of age. In the younger group, although we gave a higher dose of lapatinib, we did observe a much more robust reduction in heart weights/body weights, while lapatinib inhibited translation activation markers similarly in each model. Comparison of our two dosing models and the subsequent heart weight/body weight results may uncover novel mechanisms of hypertrophy or novel mechanisms related to ErbB2 biology.

It is tempting to speculate that the timing of lapatinib initiation is crucial and these two treatment schemes should be studied for novel ways by which ErbB2 could affect cell size or cell number. It is possible that ErbB2 transgene expression, we see dramatically induced via αMHC induction in the first week of postnatal life, has an effect on chromatin remodeling, thus influencing hypertrophy or possibly hyperplasia. When ErbB2 (and EGFR) are pharmacologically inhibited after transgene activation, potential chromatin remodeling changes may have already taken place, and may not be so easily or rapidly reversed in the 3 weeks of treatment. Future studies will be necessary to mechanistically link ErbB2 to chromatin remodeling. Yet a more plausible suggestion relates to cardiomyocyte proliferation. https://jhem.johnshopkins.edu/iframe.html - _msocom_1 ErbB2 expression in the first week of life may extend the length of time cardiomyocytes divide, thus inhibiting ErbB2 signaling at this stage may both inhibit proliferative and hypertrophic ErbB2 effects. Later in life,ErbB2’s effect on hyperplasia may not be as dominant as in neonatal period, although, recently it was found that NRG/ErbB4 pathway does induce hyperplasia in adult cardiomyocytes [49]. This hypothesis has to be tested but cardiomyocyte proliferation may explain the differences in HW/BW changes in the two lapatinib dosing models.

Furthermore, and most importantly, the present study may shed some light on why synergistic toxicity occurs in some cancer therapy patients treated with ErbB2 inhibitors (Herceptin) and doxorubicin [10]. We suggest that some patients with various stages of cardiac hypertrophy, including sub-clinical stages of disease, have dependence on ErbB2 to maintain cardiac function, and loss of ErbB2 activity (which can occur as a result of cancer therapy) results in heart failure.

## Supporting Information

Figure S1 **Kaplan-Meyer survival curves of wild type and ErbB2 transgenic mice.** Kaplan-Meyer survival curves for males (A) and females (B) of wild type (square - ▪) and ErbB2 transgenic (diamond - ♦) mouse lines. n = 142 (wild type males), n = 154 (transgenic males), n = 59 (wild type females), n = 58 (transgenic females).
(TIF)

Figure S2 **Chest radiography and gross morphology of the heart display significant cardiac hypertrophy in ErbB2 transgenic mice.** Chest anterio-posterior radiography reveals enlarged cardiac silhouette in ErbB2 transgenic mouse (B), compared to a normal size of the cardiac silhouette in wild type mouse (A). Bone structures, liver and clear lung fields are also visible in both wild type and ErbB2 transgenic mice radiographs. Wild type (C) and ErbB2 transgenic (D) hearts *in situ*. ErbB2 transgenic mouse present with enlarged heart with enlargement of both atria and ventricles. 8–10 weeks old mice were used, n = 2 per genotype.
(TIF)

Figure S3 **Heart weight and heart weight-to-body weight ratios are increased in ErbB2 transgenic mice.** Heart weights (A) and body weights (C) were measured in wild type and ErbB2 transgenic mice at P3.5, 6.5, 9.5 (n = 5–22 per genotype per age group). The data are presented as the mean ± SD. Heart weights (B), body weights (D) and heart weight-to-body weight ratios (E) were measured in wild type and ErbB2 transgenic mice at 1, 2, 4, 8, 12 months (n = 4–27 per genotype per age group). The data are presented as the mean ± SD.
(TIF)

Figure S4 **Age-related cardiac fibrosis in ErbB2 transgenic mice.** Wild type and ErbB2 transgenic hearts were examined by Masson’s trichrome staining. 2 months old wild type (A) or ErbB2 transgenic (B) mice hearts, 6 months old wild type (C) or ErbB2 transgenic (D) mice hearts, and 12 months old wild type (E) or ErbB2 transgenic (F) mice hearts were evaluated.
(TIF)

Table S1 Primers used for quantitative RT-PCR.
(DOCX)

Table S2 Buffers composition.
(DOCX)

Table S3 Antibodies used for immunoprecipitation and western blotting.
(DOCX)
