# Supplementary figures and images for: Cardiac-Specific Over-Expression of Epidermal Growth Factor Receptor 2 (ErbB2) Induces Pro-Survival Pathways and Hypertrophic Cardiomyopathy in Mice
Source: PLoS One. 2012 Aug 9;7(8):e42805. doi: 10.1371/journal.pone.0042805 (PMC3415416; doi:10.1371/journal.pone.0042805)

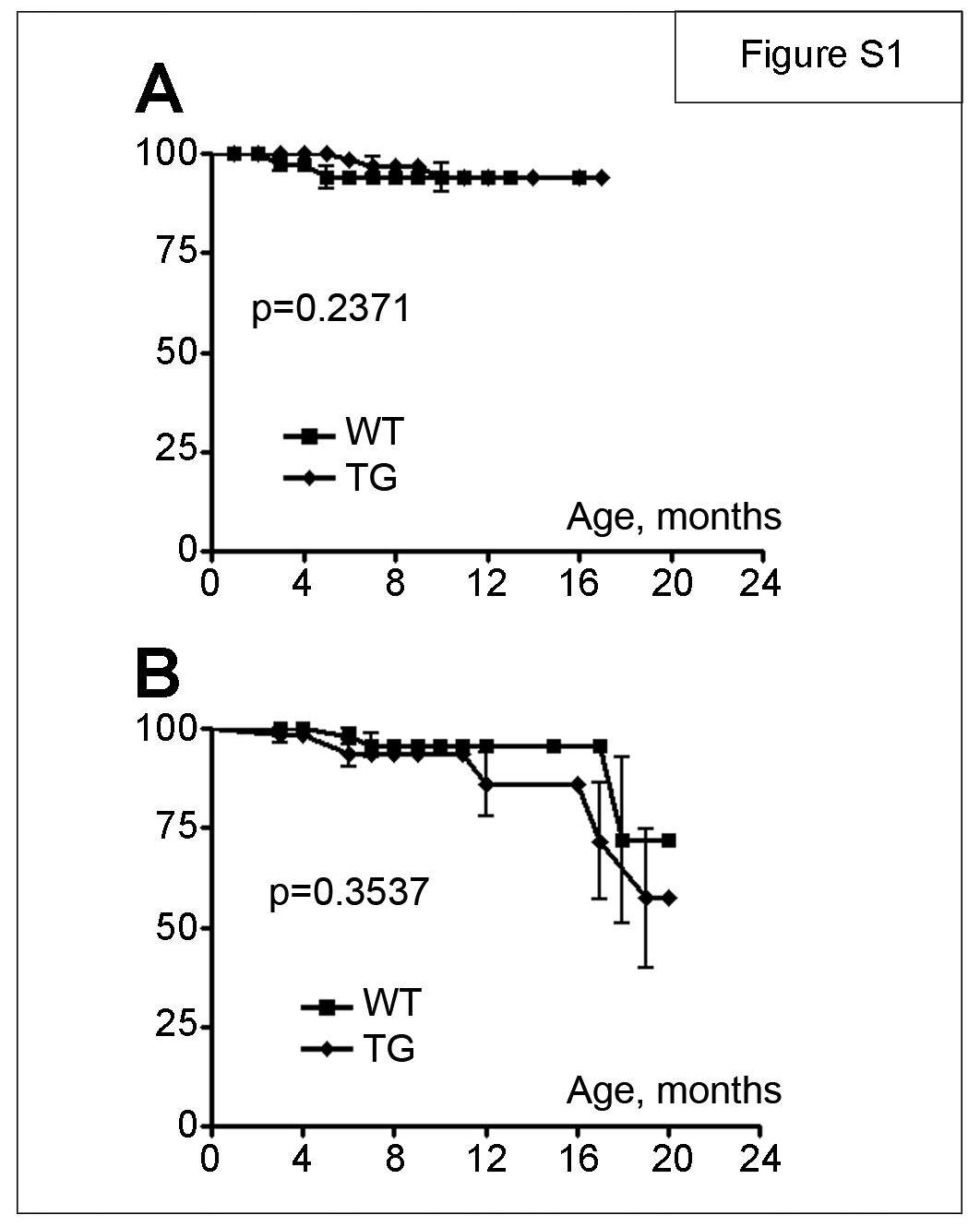

Supplement: Figure S1 — Kaplan-Meyer survival curves of wild type and ErbB2 transgenic mice. Kaplan-Meyer survival curves for males (A) and females (B) of wild type (square - ▪) and ErbB2 transgenic (diamond - ♦) mouse lines. n = 142 (wild type males), n = 154 (transgenic males), n = 59 (wild type females), n = 58 (transgenic females). (TIF) [file pone.0042805.s001.tif]

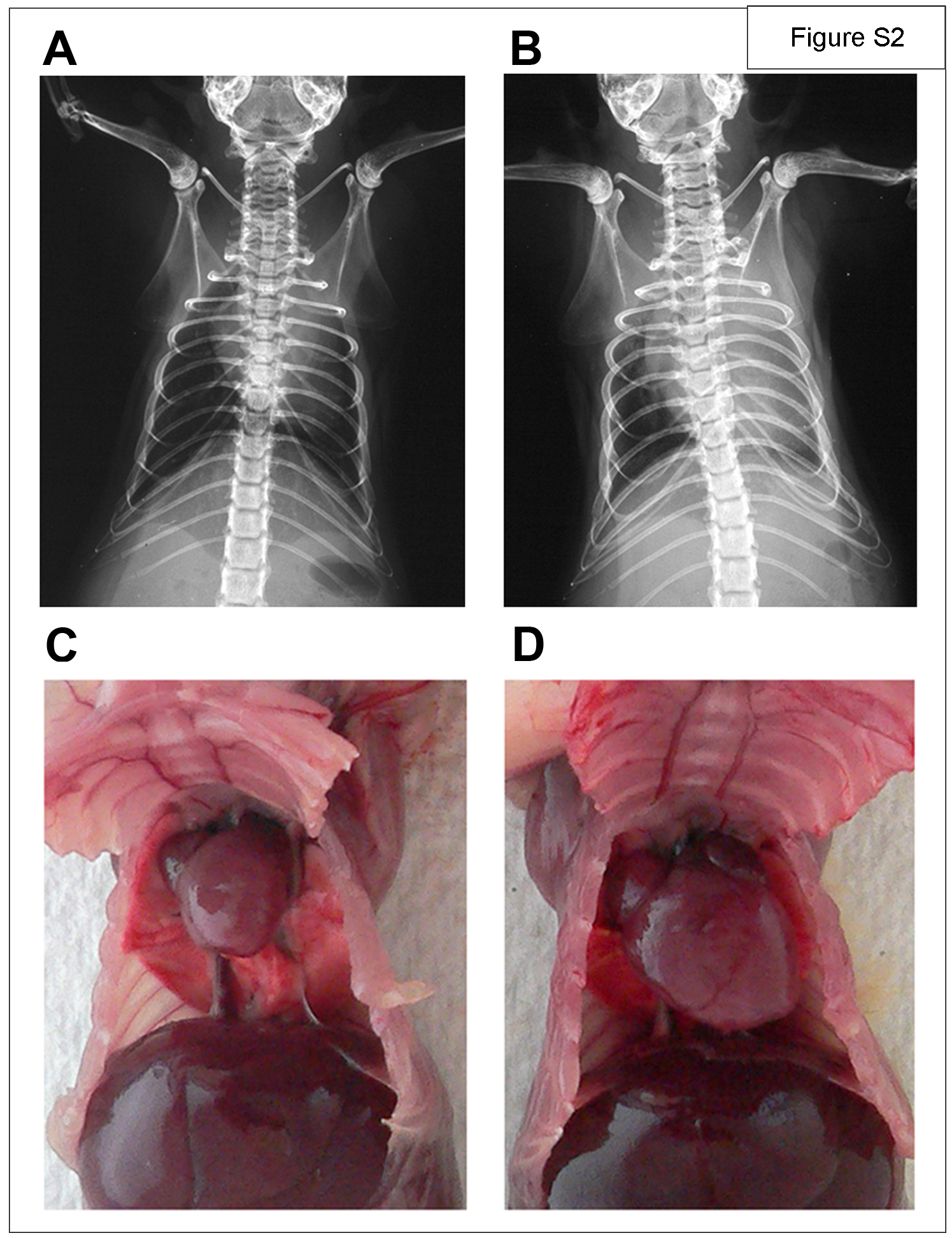

Supplement: Figure S2 — Chest radiography and gross morphology of the heart display significant cardiac hypertrophy in ErbB2 transgenic mice. Chest anterio-posterior radiography reveals enlarged cardiac silhouette in ErbB2 transgenic mouse (B), compared to a normal size of the cardiac silhouette in wild type mouse (A). Bone structures, liver and clear lung fields are also visible in both wild type and ErbB2 transgenic mice radiographs. Wild type (C) and ErbB2 transgenic (D) hearts in situ. ErbB2 transgenic mouse present with enlarged heart with enlargement of both atria and ventricles. 8–10 weeks old mice were used, n = 2 per genotype. (TIF) [file pone.0042805.s002.tif]

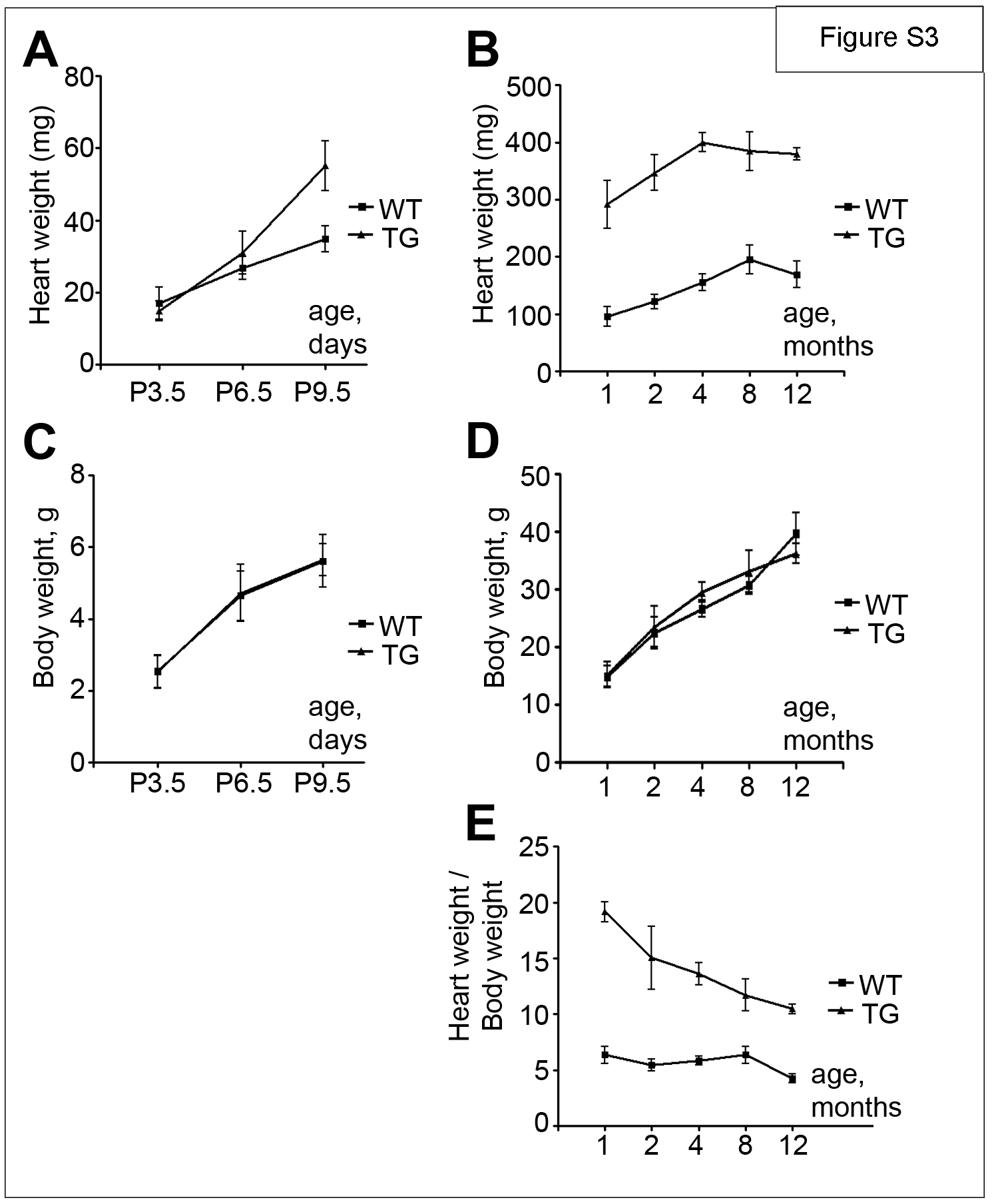

Supplement: Figure S3 — Heart weight and heart weight-to-body weight ratios are increased in ErbB2 transgenic mice. Heart weights (A) and body weights (C) were measured in wild type and ErbB2 transgenic mice at P3.5, 6.5, 9.5 (n = 5–22 per genotype per age group). The data are presented as the mean ± SD. Heart weights (B), body weights (D) and heart weight-to-body weight ratios (E) were measured in wild type and ErbB2 transgenic mice at 1, 2, 4, 8, 12 months (n = 4–27 per genotype per age group). The data are presented as the mean ± SD. (TIF) [file pone.0042805.s003.tif]

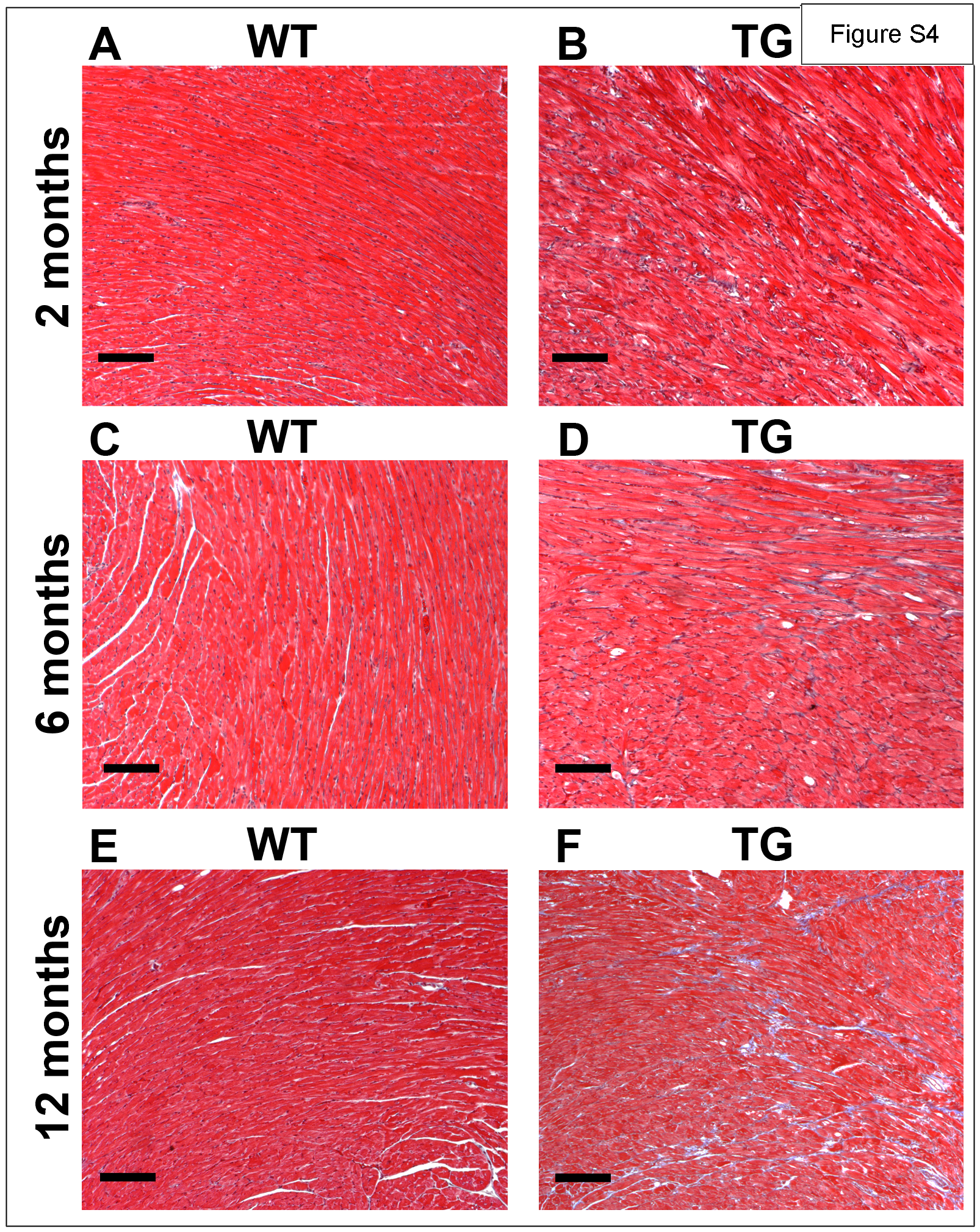

Supplement: Figure S4 — Age-related cardiac fibrosis in ErbB2 transgenic mice. Wild type and ErbB2 transgenic hearts were examined by Masson’s trichrome staining. 2 months old wild type (A) or ErbB2 transgenic (B) mice hearts, 6 months old wild type (C) or ErbB2 transgenic (D) mice hearts, and 12 months old wild type (E) or ErbB2 transgenic (F) mice hearts were evaluated. (TIF) [file pone.0042805.s004.tif]
