# Supplementary material for: Cardiac-Specific Over-Expression of Epidermal Growth Factor Receptor 2 (ErbB2) Induces Pro-Survival Pathways and Hypertrophic Cardiomyopathy in Mice
Source: PLoS One. 2012 Aug 9;7(8):e42805. doi: 10.1371/journal.pone.0042805 (PMC3415416; doi:10.1371/journal.pone.0042805)
Supplement: Table S1 — Primers used for quantitative RT-PCR. (DOCX) [file pone.0042805.s005.docx]

Table S1. Primers used for quantitative RT-PCR.

| Gene | Accession number | Sequences |
| --- | --- | --- |
| Mus musculus myosin, heavy polypeptide 7, cardiac muscle, beta | NM_080728.2 | mMYH-F 5’- TCCGAGAAAGGAAGCCTCAGCAG-3’  mMYH-R 5’- GCCACCCTCTCGGGACACGAT-3’ |
| Mus musculus natriuretic peptide type A | NM_008725.2 | mNPPA-F 5’- CAGATCGTGCCCCGACCCAC-3’  mNPPA-R 5’- GAGCTAAGTGCGGCCCCTGC-3’ |
| Peptidylprolyl isomerase A | NM_008907 | mPPIA-F 5’- GCGGCAGGTCCATCTACG-3’  mPPIA-R 5’- GCCATCCAGCCATTCAGTC-3’ |
