# Supplementary material for: Cardiac-Specific Over-Expression of Epidermal Growth Factor Receptor 2 (ErbB2) Induces Pro-Survival Pathways and Hypertrophic Cardiomyopathy in Mice
Source: PLoS One. 2012 Aug 9;7(8):e42805. doi: 10.1371/journal.pone.0042805 (PMC3415416; doi:10.1371/journal.pone.0042805)
Supplement: Table S2 — Buffers composition. (DOCX) [file pone.0042805.s006.docx]

Table S2. Buffers composition.

| RIPA | Adult cardiomyocytes isolation buffer | Immunoprecipitation buffer: | Urea sample buffer |
| --- | --- | --- | --- |
| 25mM Tris-HCl;  150mM NaCl;  1% NP-40;  1% sodiumdeoxycholate;  0.1% SDS;  1x proteinase inhibitor (Roche, cat.#11697498001); phosphatase inhibitors (Sigma, Cat.#P5726 and P0044) | 120 mM NaCl;  5.4 mM KCl;  1.2 mM NaH2PO4;  20 mM NaHCO3;  1.6 mM MgCl2;  glucose (1 mg/ml);  2,3-butanedione monoxime (BDM) 1 mg/ml;  taurine (0.628 mg/ml) | 25mM   Na2HPO4; 25mM   NaH2PO4; 150mM NaCl; 10% Glycerol; 1mM  EDTA; 1% Triton X-100; 1x proteinase inhibitor (Roche, cat.#11697498001); phosphatase inhibitors (Sigma, Cat.#P5726 and P0044) | 1% SDS 9M Urea 25mM Tris-HCl 1mM EDTA 5%  2-mercaptoethanol |
