# Supplementary material for: Cardiac-Specific Over-Expression of Epidermal Growth Factor Receptor 2 (ErbB2) Induces Pro-Survival Pathways and Hypertrophic Cardiomyopathy in Mice
Source: PLoS One. 2012 Aug 9;7(8):e42805. doi: 10.1371/journal.pone.0042805 (PMC3415416; doi:10.1371/journal.pone.0042805)
Supplement: Table S3 — Antibodies used for immunoprecipitation and western blotting. (DOCX) [file pone.0042805.s007.docx]

Table S3. Antibodies used for immunoprecipitation and western blotting.

| Protein | Vendor | Catalog # | Dilution |
| --- | --- | --- | --- |
| ErbB2 | Santa Cruz Biotechnology, Santa Cruz, CA | sc-284 | 1:1000 |
| EGFR | Santa Cruz Biotechnology, Santa Cruz, CA | sc-03 | 1:500 |
| ErbB3 | Santa Cruz Biotechnology, Santa Cruz, CA | sc-285 | 1:500 |
| ErbB4 | Santa Cruz Biotechnology, Santa Cruz, CA | sc-283 | 1:500 |
| Bcl-2 | Santa Cruz Biotechnology, Santa Cruz, CA | sc-492 | 1:1000 |
| PI3K-85 | Santa Cruz Biotechnology, Santa Cruz, CA | sc-1637 | 1:1000 |
| PI3K-110 | Santa Cruz Biotechnology, Santa Cruz, CA | sc-8010 | 1:1000 |
| AKT | Cell Signaling Technology, Danvers, MA | 9272 | 1:1000 |
| Phospho-AKT | Cell Signaling Technology, Danvers, MA | 9271 | 1:500 |
| Phospho-PTEN | Cell Signaling Technology, Danvers, MA | 9554 | 1:500 |
| PTEN | Cell Signaling Technology, Danvers, MA | 9552 | 1:1000 |
| Phospho-P70S6K | Cell Signaling Technology, Danvers, MA | 9205 | 1:500 |
| P70S6K | Cell Signaling Technology, Danvers, MA | 9202 | 1:1000 |
| Phospho-S6 | Cell Signaling Technology, Danvers, MA | 4858 | 1:500 |
| Phospho-4E-BP1 | Cell Signaling Technology, Danvers, MA | 2855 | 1:500 |
| Phospho-eIF4E | Cell Signaling Technology, Danvers, MA | 9741 | 1:500 |
| Phospho-Tyrosine | Cell Signaling Technology, Danvers, MA | 9411 | 1:500 |
| Bcl-XL/XS | BD Pharmingen, San Diego, CA | 556361 | 1:500 |
| eIF4E | BD Transduction Laboratories, San Jose, CA | 610269 | 1:1000 |
| HSP70 | Enzo Life Sciences (Stressgen), Farmingdale, NY | SPA-812 | 1:1000 |
| HSP25 | Enzo Life Sciences (Stressgen), Farmingdale, NY | SPA-801 | 1:2000 |
| HSP90 | Enzo Life Sciences (Stressgen), Farmingdale, NY | SPA-835 | 1:1000 |
| HSF1 | Cayman Chemical, Ann Arbor, MI | 10011433 | 1:500 |
